# One‐Step Thermo‐Mechanochemical Syntheses of Metal Phthalocyanines and Polyphthalocyanines

**DOI:** 10.1002/chem.202501260

**Published:** 2025-07-25

**Authors:** Stefanie Hutsch, Malte Niewind, Sven Grätz, Lars Borchardt

**Affiliations:** ^1^ Department Inorganic Chemistry Ruhr‐Universität Bochum Universitätsstraße 150 44801 Bochum Germany

**Keywords:** cobalt phthalocyanine, cobalt polyphthalocyanine, metal phthalocyanines, sustainability

## Abstract

We present a thermo‐mechanochemical synthesis of metal phthalocyanines with varying metal centers and building blocks. The reactions were performed using a Retsch Mixer Mill 400 equipped with heating jackets, achieving quantitative yields at 120 °C within 2 h. A systematic screening of reaction parameters, including temperature, reaction time, frequency, and liquid‐assisted grinding (LAG), revealed that temperature and time significantly influence the reaction outcome, as well as the use of LAG compared to neat grinding, which further enhances the yield. Powder X‐ray diffraction analysis of the obtained metal phthalocyanines confirmed the presence of different polymorphs, suggesting a correlation between reaction conditions and crystallinity. Furthermore, the crystalline cobalt polyphthalocyanine (CoPPc) was successfully synthesized with a yield of >99%.

## Graphical abstract

In this study, we report the thermo‐mechanochemical synthesis of metal phthalocyanines using a ball mill with heating jackets, achieving quantitative yields at 120 °C within 2 h. The parameter screenings revealed an insight into the reactivity of the reaction. Furthermore, various metal centers and building blocks were utilized to emphasize the variability of this approach. Lastly, a crystalline cobalt polyphthalocyanine was synthesized with >99% yield.

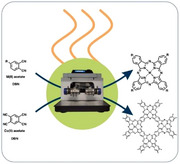

## 1 Introduction

Phthalocyanines (Pc) and their metal complexes are versatile in their chemical and physical properties, making them highly valuable across various fields of technology and science.^[^ ^1^, ^2^ ^]^ Metal phthalocyanines (MPc's) are mostly known and widely used as dyes and pigments in paints, plastics, and other materials, where their stability and vivid coloration offer distinct advantages.^[^ ^2^, ^3^ ^]^ Beyond their role as dyes, phthalocyanines and their metal complexes serve important functions in areas, among others, sensors,^[^ ^4^ ^]^ organic solar cells,^[^ ^5^ ^]^ photosensitizers for phototherapy,^[^ ^6^ ^]^ and catalysis.^[^ ^7^ ^]^ Various approaches for synthesizing different metal phthalocyanines have been studied, including techniques such as microwave irradiation^[^ ^8^ ^]^ and UV light techniques^[^ ^9^ ^]^ as well as alternative media like ionic liquids^[^ ^10^ ^]^ and solvothermal conditions^[^ ^11^, ^12^ ^]^

However, processes for synthesizing such compounds require not only harsh conditions^[^ ^11^, ^13^ ^]^ but also long reaction times.^[^ ^14^ ^]^ In response to sustainability goals and climate change considerations, the chemical industry is exploring environmentally friendly methods and processes.^[^ ^15^ ^]^ Considering these efforts, Langerreiter et al. devised a method combining pre‐milling and thermal treatment processes to synthesize various phthalocyanines.^[^ ^16^ ^]^ This approach reduces solvent consumption; however, it requires two steps and extended reaction times of 2 to 7 days.^[^ ^16^ ^]^

As indicated, the milling of chemicals can serve as an alternative to solvent‐based chemistry. Techniques such as ball milling or extrusion represent solid‐state approaches in which mechanical forces—including pressure, shock, impact, or shearing—act upon reactant particles, milling balls, or the vessel wall inside a ball mill, thereby not only mixing the reactants but also initiating the chemical reaction.^[^ ^17^ ^]^ This process, known as mechanochemistry, can play a significant role in advancing sustainable strategies. Mechanochemistry has already been applied to various reactions, such as photochemical reactions,^[^ ^18^ ^]^ catalysis,^[^ ^19^ ^]^ and material synthesis,^[^ ^20^ ^]^ aligning with the 12 principles of green chemistry.^[^ ^21^ ^]^ This approach can significantly reduce solvent waste and help avoid other harsh reaction conditions.

Since mechanical and thermal energy are interconnected and difficult to separate, it is important to investigate them in combination, known as thermo‐mechanochemistry.^[^ ^22^ ^]^ Interestingly, mechanochemical synthesis routes that were previously unsuccessful have been shown to work when temperature is applied during milling.^[^ ^23^ ^]^ The palladium‐catalyzed C‐N cross‐coupling of carbazoles and aryl halides by Kubota et al. exemplifies this integration of thermal and mechanical energy inputs, highlighting advantages such as facilitating reactions with insoluble reactants, an achievement not possible with traditional solution‐based chemistry.^[^ ^23^ ^]^ These advances in thermo‐mechanochemistry hold promise for phthalocyanine synthesis, offering new pathways for efficient and sustainable processes.

**Figure 1:**
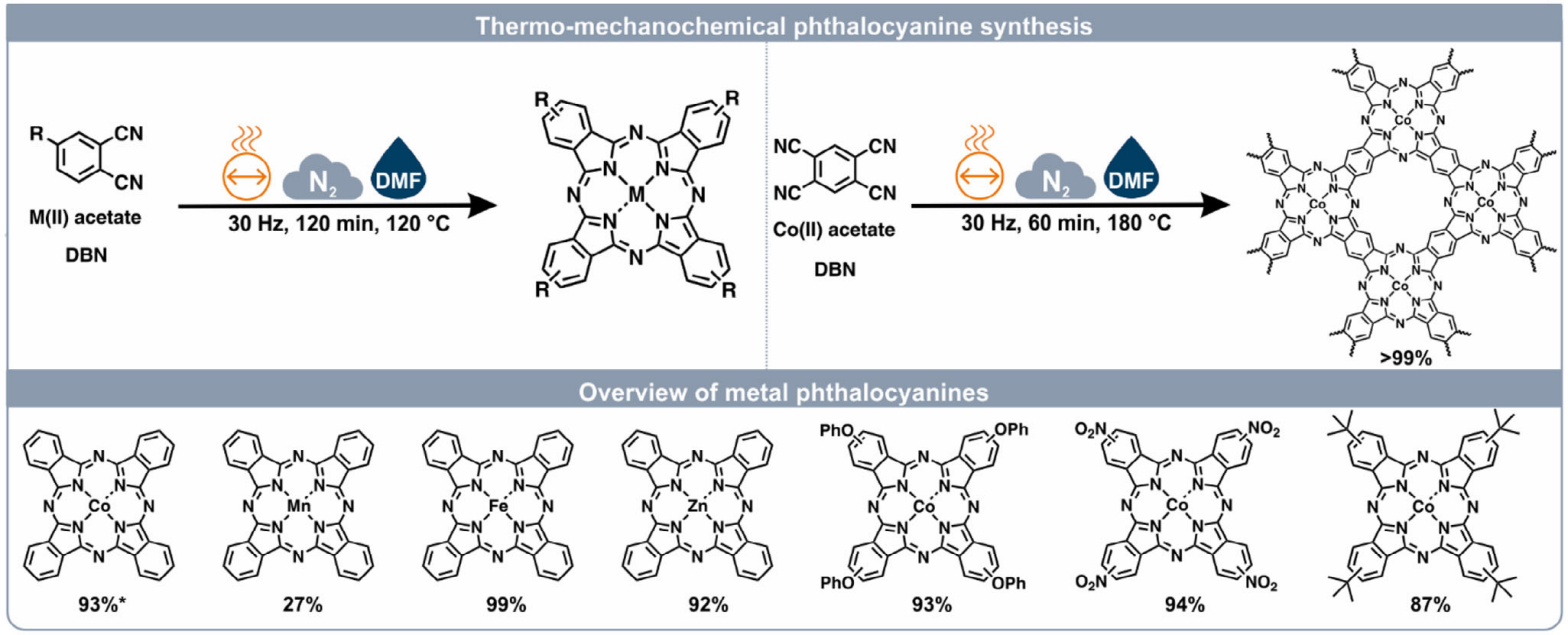
Thermo‐mechanochemical metal phthalocyanine synthesis. Top: Reaction conditions to obtain various metal phthalocyanine using a MM400 vibrational ball mill with 14 mL steel jars, one 10 mm, steel ball and heating jackets. Bottom: Reaction conditions of the cobalt phthalocyanine framework by utilize a MM400 vibrational ball mill with 14 mL steel jars, one 10 mm steel ball, and heating jackets. * 60 min reaction time.

Building on this potential, we present the one‐step syntheses of phthalocyanine metal complexes and their derivatives via thermo‐mechanochemistry. Detailed parameter screenings were executed to see the influence of this thermo‐mechanochemical procedure, aiming to gain a deeper insight. Furthermore, a dependency of the crystal structure on the mechanochemical parameter was detected. Last but not least, cobalt polyphthalocyanine (CoPPc) was obtained by optimized conditions of the standard procedure (Figure 1).

## 2 Results and Discussion

Building on nitriles as key precursors, we initiated our study by employing metal chlorides or acetates in combination with non‐nucleophilic bases such as 1,5‐diazabicyclo[4.3.0]non‐5‐ene (DBN) or 1,8‐diazabicyclo[5.4.0]undec‐7‐ene (DBU) to activate the nitrile. In preliminary experiments to synthesize Co(II)Pc, phthalonitrile (PN) and anhydrous cobalt(II) chloride were milled at 80 °C; however, no product was formed. Another reference experiment was done with cobalt(II) acetate, which failed as well, underlining the necessity of a base. For the next experiment, DBN was added to the PN and the cobalt(II) acetate, resulting in 4% of CoPc. In solvent‐based reactions,^[^ ^13^ ^]^ liquids such as *n*‐pentanol or N,N‐dimethylformamide (DMF) were used, which have now been selected as additives to increase the yield. Consequently, phthalonitrile (1.03 g, 8 mmol) and cobalt(II) acetate anhydrous (0.354 mg, 2 mmol) were combined with a small amount of DBN (69.2 µL, 0.56 mmol) and 207.6 µL DMF. This mixture (ratio 1:3) corresponded to a liquid‐assisted grinding η value of 0.2 mL mg⁻¹. The first approach was carried out in a 14 mL steel jar at 80 °C and 30 Hz for 60 min using an MM400 mixer mill. A final reference test was conducted under these conditions with the ball removed from the vessel to assess the impact of mechanical forces. The experiment resulted in a 24% yield, which is 20% lower than the reaction performed with a ball, demonstrating the mechanical contribution to the reaction.

**Figure 2:**
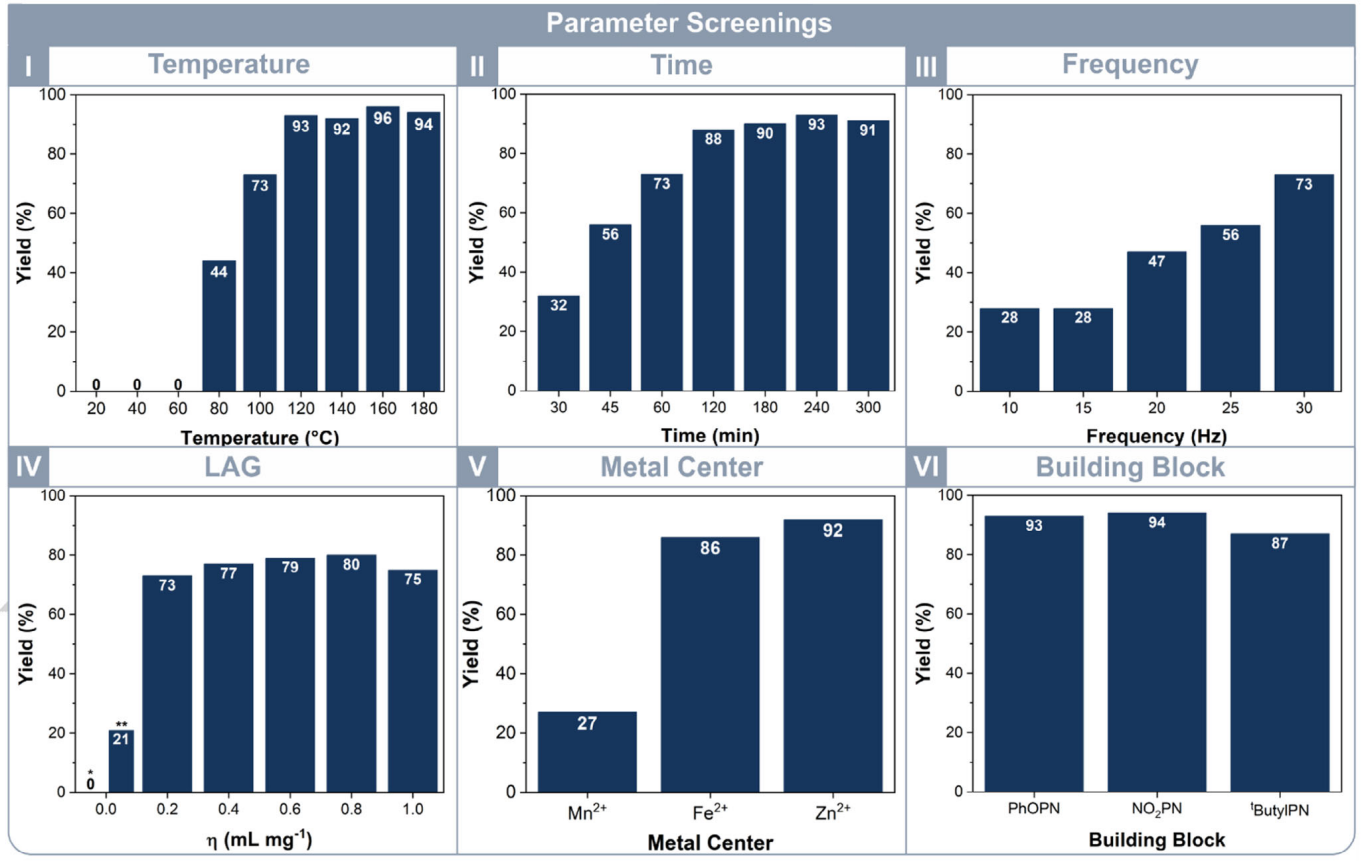
I) Temperature screening from 20 °C to up to 180 °C illustrating no reaction below 80 °C, a large step in yield from 80 to 100 °C, and a plateau for temperatures above 120 °C. II) Time screening displaying a yield increase with time until a level is reached at 120 min. III) Frequency screening illustrates increasing yield above frequencies of 20 Hz. IV) Liquid‐assisted grinding screening revealed no product formation in the absence of DBN and DMF (marked with *), a 21% yield with DBN alone (marked with **), and a significant increase at η=0.2 mL mg^−^¹. However, no further increase in yield was observed at higher η values. V) Metal center screening of the metal ions Mn^2+^, Fe^2+,^ and Zn^2+^. VI) Screening of the building blocks 4‐phenoxyphthalonitrile (PhOPN), 4‐nitrophthalonitrile (NO_2_PN), and 4‐(tert‐butyl)phthalonitrile (^t^ButylPN) at 120 °C reaction temperature for 120 min.

A comprehensive study of reaction parameters was performed to refine the conditions. The screening of reaction temperature (Figure 2, I) highlights its critical role. The synthesis of Co(II)Pc requires a minimum reaction temperature of 80 °C to initiate, as no product formation was observed at lower temperatures. Quantitative yields were achieved at temperatures exceeding 120 °C. Given that the boiling point of DBN is approximately 98 °C, its potential impact and mechanism of action at elevated temperatures require further investigation. Therefore, a reference experiment was conducted under identical conditions at 100 °C but without DBN. Instead, the DBN was replaced with an equivalent amount of DMF to maintain similar rheology. Interestingly, no reaction occurred, supporting the hypothesis that DBN is essential for initiating the reaction.

The standard parameters were set at a temperature of 100 °C; however, the same reaction conditions were maintained, including the use of the DBN to ascertain the comparability of all the reactions across surveys.

The screenings continued by investigating the influence of reaction time seen in Figure 2, II. Even after short reaction times, such as 30 min, product formation was observed. As the reaction time increased, the yield rose to 88% after 120 min, reaching a plateau. The sustainable milling and aging process of Langerreiter et al. achieved a 99% conversion at 100 °C; however, it required an aging time of 2 days. In contrast, our approach achieved similar yields at the same reaction temperature, with a time savings of over 90%, highlighting the efficiency of this rapid one step method. The effect of frequency followed the expected trend, with an increase in frequency (Figure 2, III) enhancing the reaction yield. Additionally, the impact of liquid‐assisted grinding (LAG) was investigated by varying the total amount of DBN and DMF while maintaining a constant 1:3 ratio. LAG is a promising approach usually used to enhance yield by using small amounts of liquid to accelerate the reaction. As mentioned before, using no DBN nor DMF led to no reaction at all. Using just DBN and no DMF at 100 °C, resulted in 21% yield. Interestingly, increasing the amount of the LAG‐mixture did not affect the product yield at all, suggesting that additional liquid had no impact on the reaction.

Furthermore, the applicability and versatility of the reaction were evaluated by employing various metal centers (Figure 2, V) and different building blocks (Figure 2, VI). Commonly used metal centers in phthalocyanine chemistry include manganese, iron, and zinc. Consequently, the reaction was conducted using the corresponding anhydrous metal(II) acetates at 120 °C for 120 min. FePc and ZnPc were obtained in quantitative yields. However, the yield of MnPc deviated significantly, reaching only 27%, which could potentially be improved by extending the reaction time and/or increasing the temperature.

In addition, the building block was varied to investigate the influence of different electronic effects. Functional groups were selected based on their inductive (I) and mesomeric (M) effects, including 4‐phenoxyphthalonitrile (−I, +M effect), 4‐nitrophthalonitrile (−I, ‐M effect), and 4‐(tert‐butyl)phthalonitrile (+I effect). The reactions were carried out under identical conditions at 120 °C for 120 min. The yields of the obtained products showed only little variation, with 93% for MPc_Co‐PhOPN, 94% for MPC_Co‐NO_2_PN, and 87% for MPc_Co‐^t^BuPN, suggesting that the substituent had no significant impact on the reaction outcome. Using milder conditions, set as the standard reaction parameters of 100 °C and 60 min, yielded different results. The yield for the ^t^ButylPN building block (MPc_Co‐^t^BuPN_m) was 24%, lower than that of NO_2_PN (35%) and PhOPN (47%), as seen in Figure S30.

**Figure 3:**
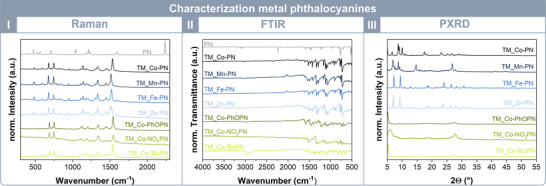
Characterization of the obtained metal phthalocyanines: I) Raman spectroscopy shows the vanishing of the C≡N vibration at 2238 cm^−1^ and the formation of the aromatic ring system at 1520 cm^−1^ (ν(C‐C_arom._)). II) FTIR spectroscopy illustrate the missing C≡N. III) The powder xray diffractions display the polymorphism of the metal phthalyocyanines, which alter between α, π and the β polymorph.

Raman and Fourier transform infrared (FTIR) analysis (Figure 3, I and II) were performed to confirm the successful product formation. In Raman spectra, the band 2238 cm^−1^ corresponding to the nitrile group, disappeared completely, indicating successful cyclotetramerization to phthalocyanine. Additionally, the Raman shift ν(C‐C_arom._) of the aromatic ring system appears at approximately 1540 cm^−1^, further confirming the formation of the target structure. Similarly, the FTIR analysis shows the disappearance of the 2220 cm^−1^ band associated with the CN group, emphasizing complete reaction.

Metal‐free and metal phthalocyanines are known to exist in various polymorphic forms, including for instance, the metastable α‐form, the stable β‐polymorph, or the intermediate π‐form. The π–π interactions between the aromatic rings and neighboring atoms can vary, influencing the tilt angle of the molecular planes, resulting in different stacking arrangements. Consequently, the reflections may shift depending on the metal center or building block. Therefore, the crystallinity of the synthesized metal phthalocyanines was analyzed, revealing the presence of different polymorphs. The PXRD pattern of MPc_Co‐PN displays π‐form reflections at 2θ = 5.0°, 8.7° and 10°.^[^ ^24^ ^]^ The metastable α‐polymorph was identified in MPc_Co‐PhOPN, MPC_Co‐NO_2_PN, MPc_Co‐^t^BuPN, each exhibiting a distinct reflection observed at approximately 2θ = 5.0–5.2°. The β‐form was observed for MPc_Fe‐PN and MPc_Zn‐PN, characterized by two reflections at 2θ = 7.2° and 9.4° for MPc_Fe‐PN and for MPc_Zn‐PN at 7.0° and 9.2° according to the literature.^[^ ^25^ ^]^ In contrast, the PXRD pattern of MPc_Mn‐PN indicates a mixture of polymorphs exhibiting two prominent reflections at 2θ = 6.9° and 8.8°, corresponding to the β‐form, along with signals at 2θ = 26.8° associated with the α polymorph.^[^ ^26^ ^]^ It is known that ball milling can control the polymorphism of metal‐free phthalocyanine through dry or wet milling.^[^ ^27^ ^]^ To examine this effect more thoroughly, different reaction parameters were examined for the obtained CoPcs. A comparison of PXRD patterns for products synthesized at 180 °C (MPc_Co‐PN_180 °C), with a reaction time of 300 min (MPc_Co‐PN_300 min), and an η value of 1 mL mg⁻¹ (MPc_Co‐PN_η1) revealed distinct polymorphic outcomes. Both MPc_Co‐PN_300 min and MPc_Co‐PN_η1 exclusively exhibited the π‐form of CoPc, similar to the standard reaction conditions (60 min, η = 0.2 mL mg⁻¹). Notably, only an increase in temperature to 180 °C led to the formation of the β‐polymorph, as shown in Figures S14–S16.

Given the ecological advantages of mechanochemistry over conventional methods, a green chemistry metrics analysis was conducted. This included calculating the E‐factor, mass intensity, and EcoScale, and comparing these values to those of alternative CoPc synthesis routes, as presented in the Supporting Information, **Chapter 6: Green Chemistry Metrics**, conclusively affirming the thermo‐mechanochemical approach as a more sustainable choice and underlining the environmental benefits of our synthesis route.

**Figure 4:**
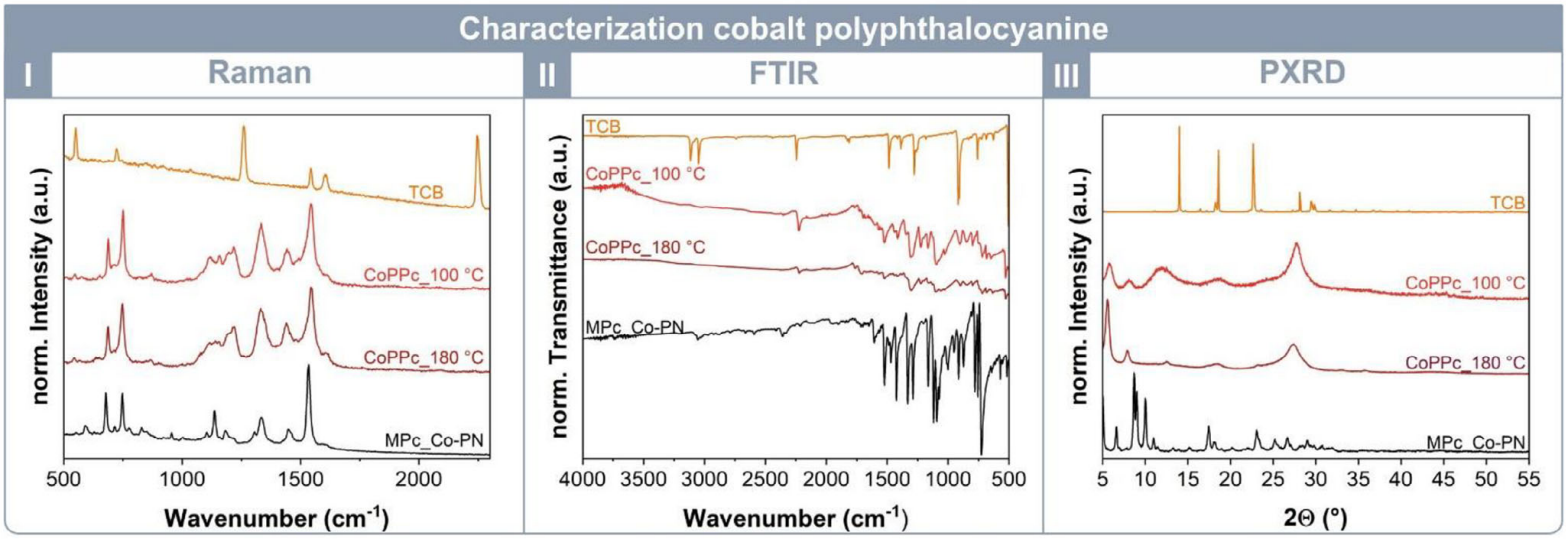
Characterization of the cobalt polyphthalocyanine (CoPPc): I) Raman spectroscopy illustrates the vanishing of the nitrile vibration and the differences between the cobalt polyphthalocyanine (dark red) and the cobalt phthalocyanine (black). II) FTIR spectroscopy shows the disappearance of the C≡N vibration to confirm the cyclotetramerization as well. III) Powder x‐ray diffractions display a crystalline cobalt polyphthalocyanine with reflections at 2θ = 5.6°, 8.0° and 27.2°.

Lastly, 1,2,4,5‐tetracyanobenzene was used as a building block to explore the potential synthesis of the cobalt polyphthalocyanine. Under the standard reaction conditions of 100 °C and a reaction time of 60 min, a deep black powder with a yield of 25% was obtained. However, FTIR analysis still displayed the C≡N stretching band, indicating incomplete conversion to the desired framework (Figure 4, II). Given the significant influence of temperature observed in previous screenings, the reaction temperature was subsequently increased to promote complete cyclotetramerization. Repeating the reaction at 180 °C resulted in a crystalline cobalt polyphthalocyanine with a yield >99%. Characterization was performed similarly to the previously analyzed MPcs. Raman spectroscopy confirmed the disappearance of the nitrile band and a strong ν(C‐C_arom._) band, consistent with FTIR observations (Figure 4, I and II). PXRD analysis revealed a crystalline CoPPc with prominent reflections at 2θ = 5.6°, 8.0°, 14.0 and 27.2° in agreement with literature.^[^ ^28^ ^]^ Alternative methods for synthesizing cobalt polyphthalocyanine include an ionothermal approach at 400 °C for 24 h^[^ ^29^ ^]^ and a solvent‐based method using ethylene glycol, which lowers the reaction temperature to 180 °C but requires a prolonged reaction time of 72 h.^[^ ^30^ ^]^ A microwave‐assisted approach at a similar temperature reduces the time to 2.5 h.^[^ ^31^ ^]^ In contrast, the ball milling protocol significantly shortens the reaction time while yielding a crystalline powder, presenting a promising approach for synthesizing other metal polyphthalocyanines.

## 3 Conclusion

In summary, we present the one‐step thermo‐mechanochemical syntheses of various crystalline metal phthalocyanines and a porous cobalt phthalocyanine framework using a ball mill equipped with heating jackets. A detailed screening containing temperature, reaction time, frequency, and liquid‐assisted grinding provided valuable insights into this process. A minimum reaction temperature of 80 °C was required for product formation, while reactions conducted at 100 °C yielded products within just 30 min, demonstrating the rapid nature of the process. Notably, DBN was essential for initiating the reaction, while the addition of DMF through liquid‐assisted grinding further enhanced the yield.

Furthermore, this one step method proved to be versatile, as demonstrated by the successful synthesis of various metal‐centered and substituted phthalocyanines. PXRD analysis revealed polymorphism in the obtained MPcs influenced by the reaction parameters. At 80 °C, the π‐form was predominantly obtained, while the more thermodynamically stable β‐form was favored at a temperature of 180 °C. This emphasizes the ability to tailor the crystallinity of the resulting metal phthalocyanines of this rapid synthesis approach.

Finally, the synthesis of the cobalt polyphthalocyanine was executed with adjusted reaction conditions of 60 min at 180 °C resulting in a crystalline black powder of >99% yield, which introduces a novel fast synthesis of metal polyphthalocyanine.

## Supporting Information

The authors have cited additional references within the Supporting Information.^[^ ^32^ ^]^

## Conflict of Interest

The authors declare no conflicts of interest.

## Supporting information



Supporting Information

## Data Availability

The data that support the findings of this study are available in the supplementary material of this article.
