# Supplementary material for: One‐Step Thermo‐Mechanochemical Syntheses of Metal Phthalocyanines and Polyphthalocyanines
Source: Chemistry. 2025 Jul 25;31(51):e01260. doi: 10.1002/chem.202501260 (PMC12434449; doi:10.1002/chem.202501260)
Supplement: Supplementary file 1 — Supporting Information [file CHEM-31-e01260-s001.pdf]

# Table of Content

1. Materials ........ 3

2. Methods ........ 4

3. Synthetic Procedures ........ 5

4. Characterization ........ 7

5. Supplementary Figures ........ 21

6. Green Chemistry Metrics ........ 22

   6.1. E-Factor ........ 22

   6.2. Mass intensity ........ 23

   6.3. EcoScale ........ 24

   6.4. Comparison Green Merics ........ 24

7. Author contributions ........ 25

8. References ........ 25

# 1. Materials

The chemicals used for the syntheses were bought from commercial sources and were not further purified: Phthalonitrile (TCI; ≥99%), Co(II)acetate ($Co(OAc)_2$, Thermo Fischer; 98%), 1,5-Diazabicyclo[4.3.0]non-5-en (DBN, Sigma-Aldrich; 98%), N,N-Dimethylformamide, anhydrous (DMF, Sigma-Aldrich; 99.8%), Mn(II)acetate (Sigma-Aldrich; 98%), Fe(II)acetate (BLD; 98%), Zn(II)acetate (Thermo Fischer; 98%), 4-Phenoxyphthalonitrile (PhOPN, BLD, 97 % purity), 4-Nitrophthalonitrile ($NO_2PN$; BLD, 98 % purity), 4-(tert-Butyl)phthalonitrile ($^{t}$butylPN, BLD, 97 % purity), and 1,2,4,5-Tetracyanobenzene (TCB, Sigma-Aldrich, 97 % purity).

Steel milling balls in Ø=10 mm with an average weight of 4.3 g were earned from Kugel Winnie. Zirconium oxide milling balls (Type ZY-S) in Ø = 10 mm with an average weight of 3.2 g were earned from Sigmund Lindner GmbH.

The air and moisture sensitive reactants were stored in a glovebox and reactions performed under inert gas were filled in the glovebox.

# 2. Methods

The Raman measurements were performed by a RENISHAW inVia Qontor Raman microscope with a 50x objective. The used wavelength was 785 nm and the exposure time was 1 s.

Fourier transform infrared (FTIR) spectroscopy was executed by a SHIMADZU IRSpirit Fourier transform infrared spectrometer equipped with a QATR-S ATR unit. The spectra were recorded with 20 scans between 500 $cm^{-1}$ and 4000 $cm^{-1}$.

Powder X-ray Diffraction (PXRD) was investigated with a Bruker D2 PHASER spectrometer with CuKα (1.54184 Å) radiation. The observed range included 5–55 ° 2θ using a 0.4 mm divergence slit.

Matrix assisted laser desorption ionization time of flight mass spectroscopy (MALDI-TOF) was executed by a Bruker Ultraflex 3.

The $^{1}H$ and $^{13}C$ NMR spectroscopy was performed using a Bruker Avance III HD spectrometer at 400 MHz. The chemical shift δ was given as parts per million (ppm).

To analyze the porosity, nitrogen adsorption measurements using high-purity gas ($N_2$: 99.999 %) were performed by the Quantachrome QuadraSorb at 77 K. Specific surface areas ($SSA_{BET}$) were calculated using the Brunauer, Emmet, and Teller (BET) equation. Before the measurement, all samples had to be activated at 353 K under vacuum for 24 h. In addition, argon, adsorption measurements were performed on a Quantachrome Autosorb instrument at 86.7 K and 298 K. Therefore, the samples were prepared similarly to those above.

# 3. Synthetic Procedures

Under a $N_2$ atmosphere, 1.03 g (8 mmol) of phthalonitrile and 0.354 mg (2 mmol) cobalt(II) acetate anhydrous were combined with a small amount of 69.2 µl (0.56 mmol) DBN and 207.6 µl DMF. This mixture corresponded to a liquid-assisted grinding η value of 0.2 ml $mg^{-1}$. The reaction was carried out in a 14 ml steel jar at 80 °C and 30 Hz for 60 minutes using an MM400 mixer mill. After the reaction and the jar cooled down 4 mL of water were added, and the mixture was milled again for 3 min at 30 Hz. The suspension was then transferred to a 50 mL falcon tube, filled with water to 50 mL, shaken thoroughly, centrifuged and decanted. The same procedure was repeated twice with acetone. The gained product was dried overnight at room temperature.

The same procedure was used for the temperature, time, frequency and liquid-assisted grinding (LAG) screening, varying these parameters.

Furthermore, the reaction parameters 120 °C and 120 min was used for the screening of the different metal acetates and building blocks.

The cobalt polyphthalocyanine synthesis was executed analogue to the approach above, exchanging the phthalonitrile to 1,2,4,5-tetracyanobenzene, reducing the time to 60 min and increase the temperature to 180 °C. The obtained deep black powder was washed with water through a Buchner funnel, subsequently washed by Soxhlet with acetone and finally was dried over night at 80 °C.

**Table S1**:Parameters of the conducted reactions. All reactions were performed in a 14 mL steel vessel with a 10 mm steel ball using a MM400 mixer mill. Reaction marked with asterix was conducted in a 14 mL PFA vessel with a 10 mm Zirconium oxide ball using a MM400 mixer mill

| Entry | Sample | Building block | Metal acetate | Base | Additive | Temperature (°C) | Time (min) | Frequency (Hz) | LAG (mL mg$^{-1}$) | Yield (%) |
|---|---|---|---|---|---|---|---|---|---|---|
| **1** | Reference 1 | PN | Co(OAc)$_2$ | - | - | 80 | 60 | 30 | - | - |
| **2** | Reference 2 | PN | Co(OAc)$_2$ | DBN | - | 80 | 60 | 30 | - | 4 |
| **3** | Reference 3 | PN | Co(OAc)$_2$ | - | DMF | 100 | 60 | 30 | 0.2 | - |
| **4** | Reference w/o ball | PN | Co(OAc)$_2$ | DBN | DMF | 100 | 60 | 30 | 0.2 | 24 |
| **5** | MPc_Co-PN_20°C | PN | Co(OAc)$_2$ | DBN | DMF | 20 | 60 | 30 | 0.2 | 0 |
| **6** | MPc_Co-PN_40°C | PN | Co(OAc)$_2$ | DBN | DMF | 40 | 60 | 30 | 0.2 | 0 |
| **7** | MPc_Co-PN_60°C | PN | Co(OAc)$_2$ | DBN | DMF | 60 | 60 | 30 | 0.2 | 0 |
| **8** | MPc_Co-PN_80°C | PN | Co(OAc)$_2$ | DBN | DMF | 80 | 60 | 30 | 0.2 | 44 |
| **9** | MPc_Co-PN_stand | PN | Co(OAc)$_2$ | DBN | DMF | 100 | 60 | 30 | 0.2 | 73 |
| **10** | MPc_Co-PN_120°C | PN | Co(OAc)$_2$ | DBN | DMF | 120 | 60 | 30 | 0.2 | 93 |
| **11** | MPc_Co-PN_140°C | PN | Co(OAc)$_2$ | DBN | DMF | 140 | 60 | 30 | 0.2 | 92 |
| **12** | MPc_Co-PN-160°C | PN | Co(OAc)$_2$ | DBN | DMF | 160 | 60 | 30 | 0.2 | 96 |
| **13** | MPc_Co-PN_180°C | PN | Co(OAc)$_2$ | DBN | DMF | 180 | 60 | 30 | 0.2 | 94 |
| **14** | MPc_Co-PN_30min | PN | Co(OAc)$_2$ | DBN | DMF | 100 | 30 | 30 | 0.2 | 32 |
| **15** | MPc_Co-PN_45min | PN | Co(OAc)$_2$ | DBN | DMF | 100 | 45 | 30 | 0.2 | 56 |
| **16** | MPc_Co-PN_120min | PN | Co(OAc)$_2$ | DBN | DMF | 100 | 120 | 30 | 0.2 | 88 |
| **17** | MPc_Co-PN_180min | PN | Co(OAc)$_2$ | DBN | DMF | 100 | 180 | 30 | 0.2 | 90 |
| **18** | MPc_Co-PN_240min | PN | Co(OAc)$_2$ | DBN | DMF | 100 | 240 | 30 | 0.2 | 93 |
| **19** | MPc_Co-PN_300min | PN | Co(OAc)$_2$ | DBN | DMF | 100 | 300 | 30 | 0.2 | 91 |
| **20** | MPc_Co-PN_10Hz | PN | Co(OAc)$_2$ | DBN | DMF | 100 | 60 | 10 | 0.2 | 28 |
| **21** | MPc_Co-PN_15Hz | PN | Co(OAc)$_2$ | DBN | DMF | 100 | 60 | 15 | 0.2 | 28 |
| **22** | MPc_Co-PN_20Hz | PN | Co(OAc)$_2$ | DBN | DMF | 100 | 60 | 20 | 0.2 | 47 |
| **23** | MPc_Co-PN_25Hz | PN | Co(OAc)$_2$ | DBN | DMF | 100 | 60 | 25 | 0.2 | 56 |
| **24** | MPc_Co-PN_neat | PN | Co(OAc)$_2$ | DBN | DMF | 100 | 60 | 30 | 0.0 | 0 |
| **25** | MPc_Co-PN_neat+DBN | PN | Co(OAc)$_2$ | DBN | DMF | 100 | 60 | 30 | | 21 |
| **26** | MPc_Co-PN_η0.4 | PN | Co(OAc)$_2$ | DBN | DMF | 100 | 60 | 30 | 0.4 | 77 |
| **27** | MPc_Co-PN_η0.6 | PN | Co(OAc)$_2$ | DBN | DMF | 100 | 60 | 30 | 0.6 | 79 |
| **28** | MPc_Co-PN_η0.8 | PN | Co(OAc)$_2$ | DBN | DMF | 100 | 60 | 30 | 0.8 | 80 |
| **29** | MPc_Co-PN_η1 | PN | Co(OAc)$_2$ | DBN | DMF | 100 | 60 | 30 | 1 | 75 |
| **30** | MPc_Mn-PN | PN | Mn(OAc)$_2$ | DBN | DMF | 120 | 120 | 30 | 0.2 | 27 |
| **31** | MPc_Fe-PN | PN | Fe(OAc)$_2$ | DBN | DMF | 120 | 120 | 30 | 0.2 | 86 |
| **32*** | MPc_Zn-PN | PN | Zn(OAc)$_2$ | DBN | DMF | 120 | 120 | 30 | 0.2 | 92 |
| **33** | MPc_Co-PhOPN | PhOPN | Co(OAc)$_2$ | DBN | DMF | 120 | 120 | 30 | 0.2 | 93 |
| **34** | MPc_Co-NO$_2$PN | NO$_2$PN | Co(OAc)$_2$ | DBN | DMF | 120 | 120 | 30 | 0.2 | 94 |
| **35** | MPc_Co-$^{t}$butylPN | $^{t}$butylPN | Co(OAc)$_2$ | DBN | DMF | 120 | 120 | 30 | 0.2 | 87 |
| **36** | MPc_Co-PhOPN_m | PhOPN | Co(OAc)$_2$ | DBN | DMF | 100 | 60 | 30 | 0.2 | 93 |
| **37** | MPc_Co-NO$_2$PN_m | NO$_2$PN | Co(OAc)$_2$ | DBN | DMF | 100 | 60 | 30 | 0.2 | 94 |
| **38** | MPc_Co- $^{t}$butylPN_m | $^{t}$butylPN | Co(OAc)$_2$ | DBN | DMF | 100 | 60 | 30 | 0.2 | 87 |
| **39** | CoPPc_100°C | TCB | Co(OAc)$_2$ | DBN | DMF | 100 | 60 | 30 | 0.2 | 25 |
| **40** | CoPPc_180°C | TCB | Co(OAc)$_2$ | DBN | DMF | 180 | 120 | 30 | 0.2 | 99 |

# 4. Characterization

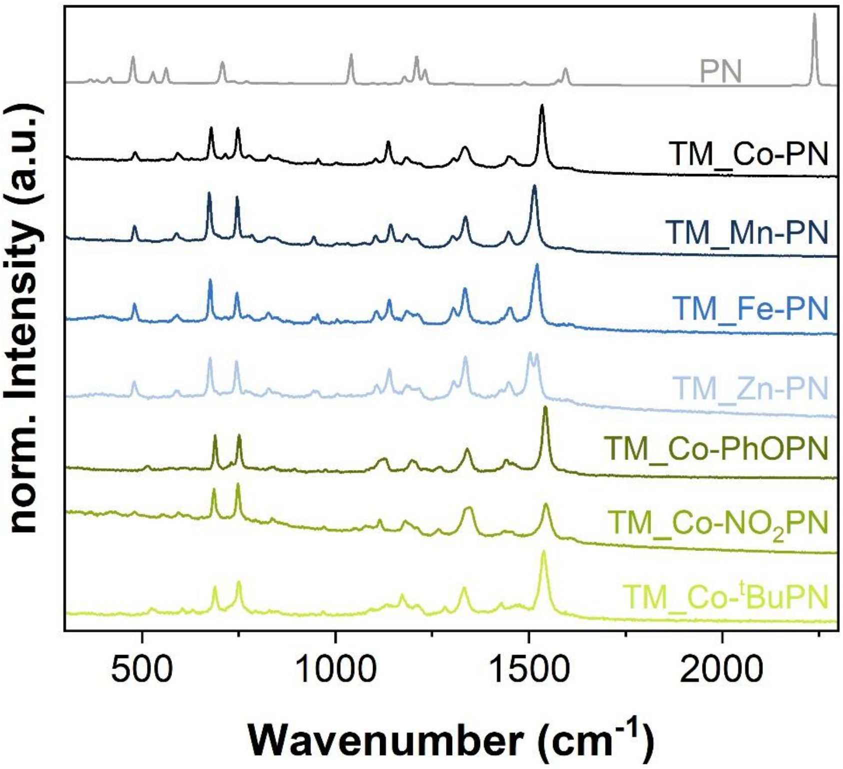


**Figure S1**: Raman spectroscopy of the obtained metal phthalocyanines.

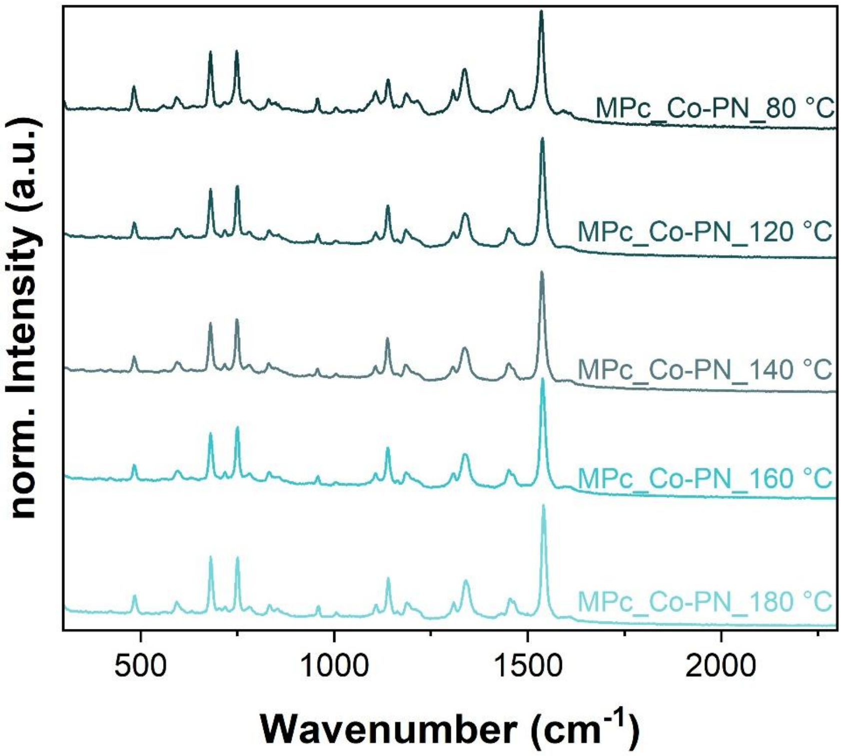


**Figure S2**: Raman spectroscopy of the obtained cobalt phthalocyanines of the temperature screening.

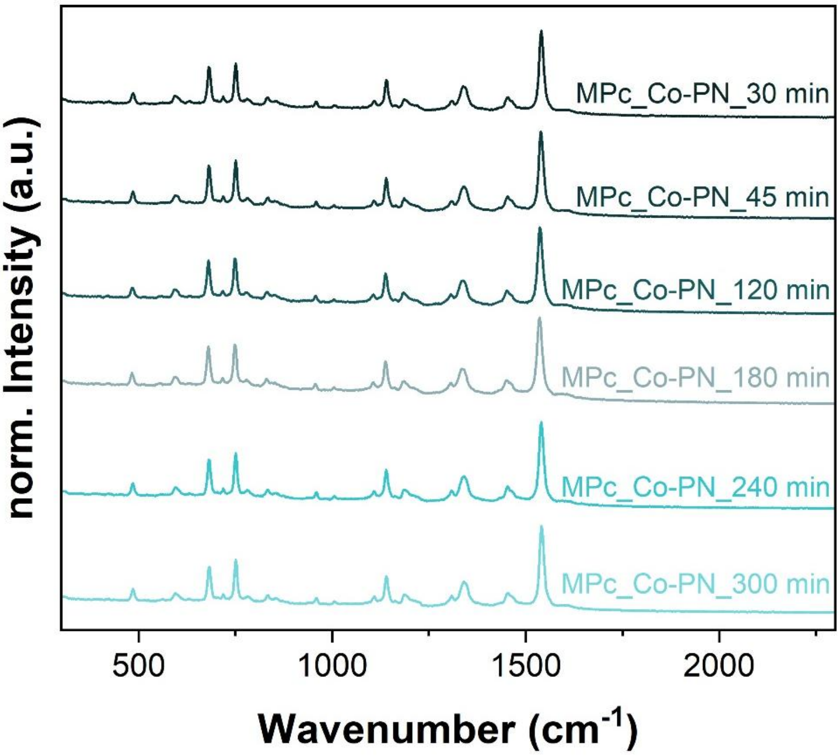


**Figure S3**: Raman spectroscopy of the obtained cobalt phthalocyanines of the time screening.

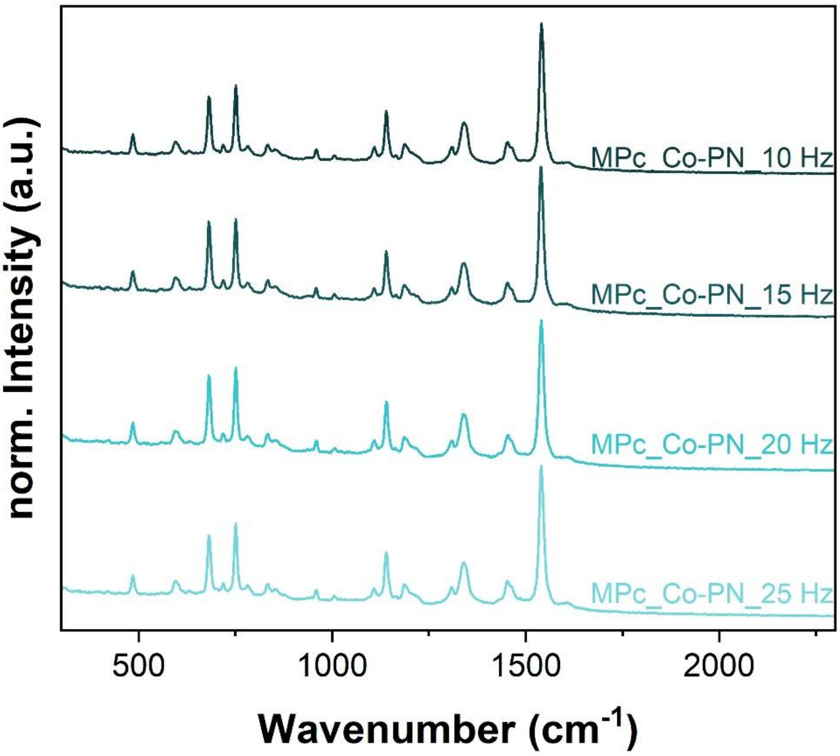


**Figure S4**: Raman spectroscopy of the obtained cobalt phthalocyanines of the frequency screening.

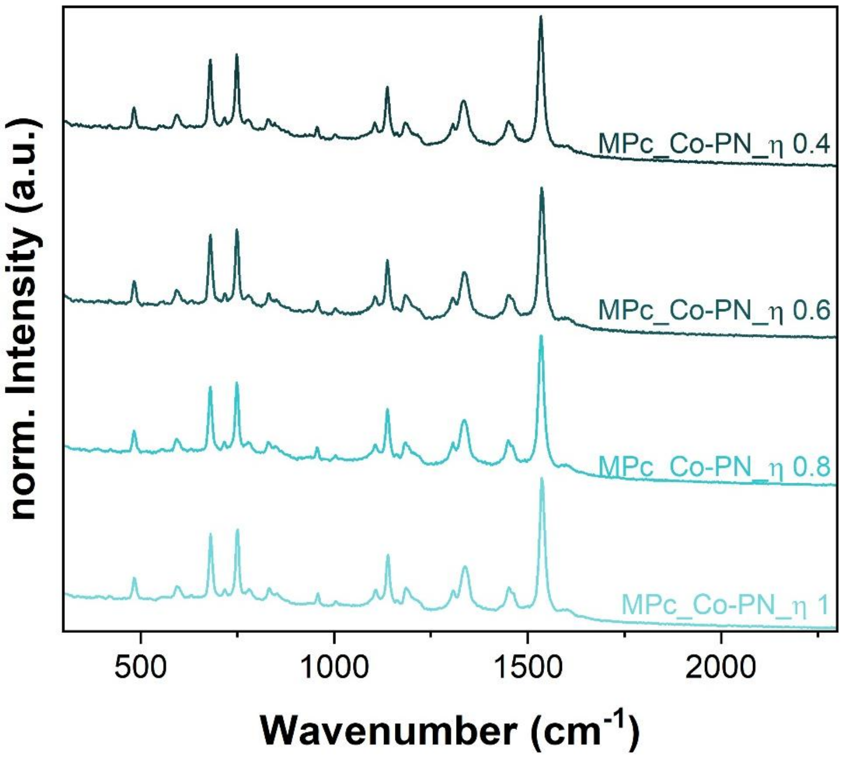


**Figure S5**: Raman spectroscopy of the obtained cobalt phthalocyanines of the liquid-assisted grinding screening.

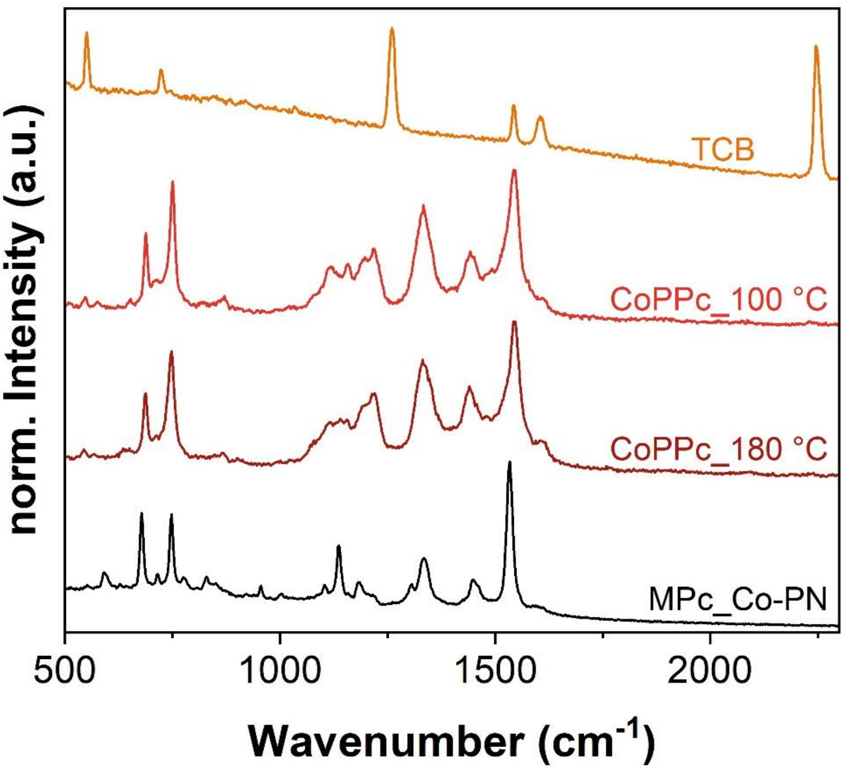


**Figure S6**: Raman spectroscopy of the obtained cobalt polyphthalocyanine.

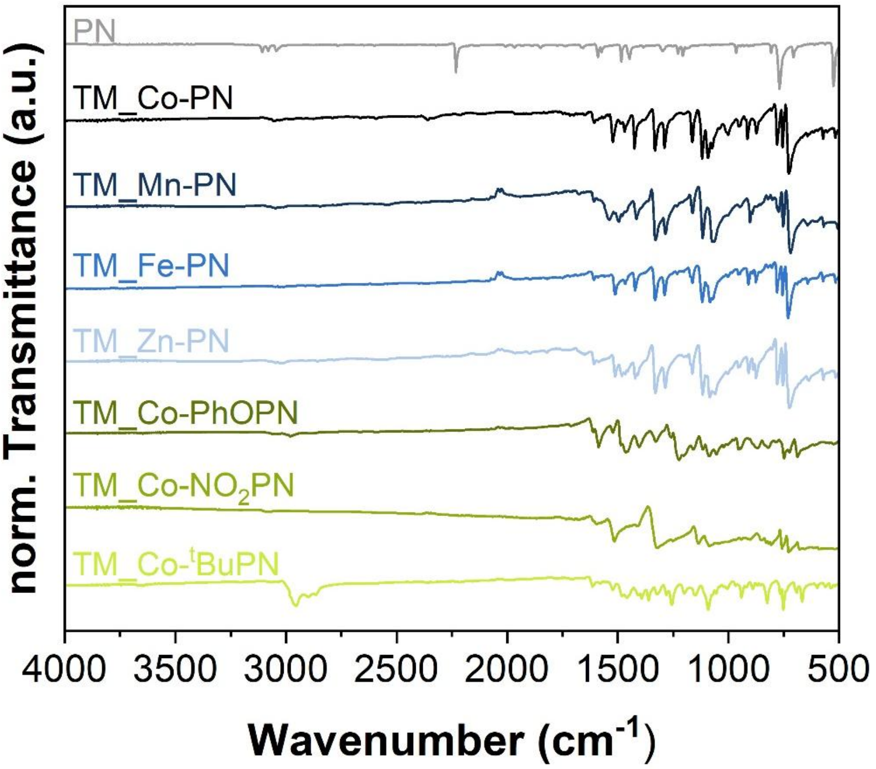


**Figure S7**: FTIR spectroscopy of the obtained metal phthalocyanines.

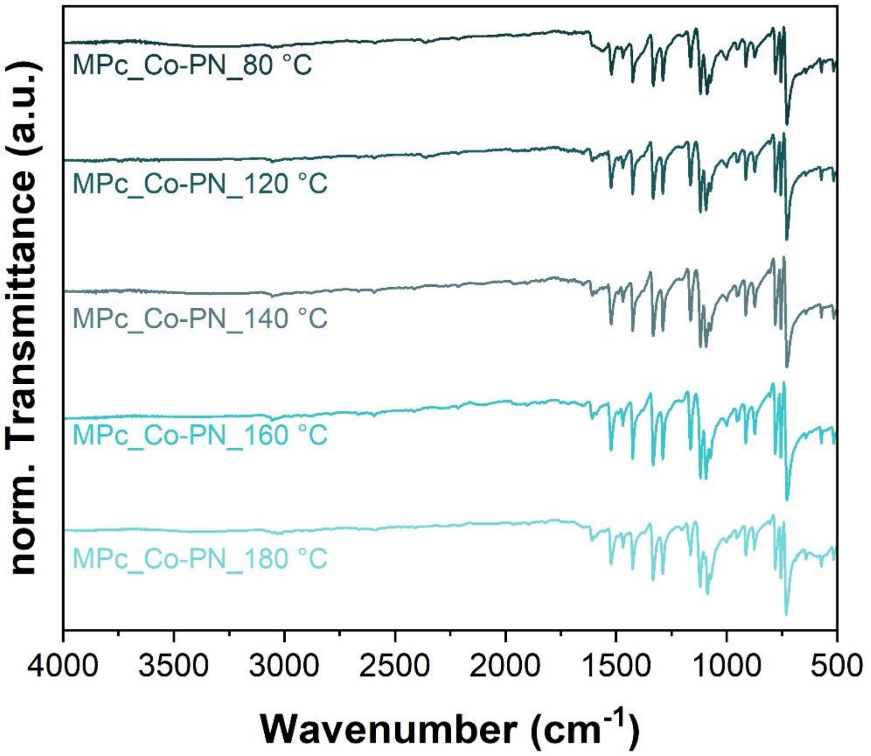


**Figure S8**: FTIR spectroscopy of the obtained cobalt phthalocyanines of the temperature screening.

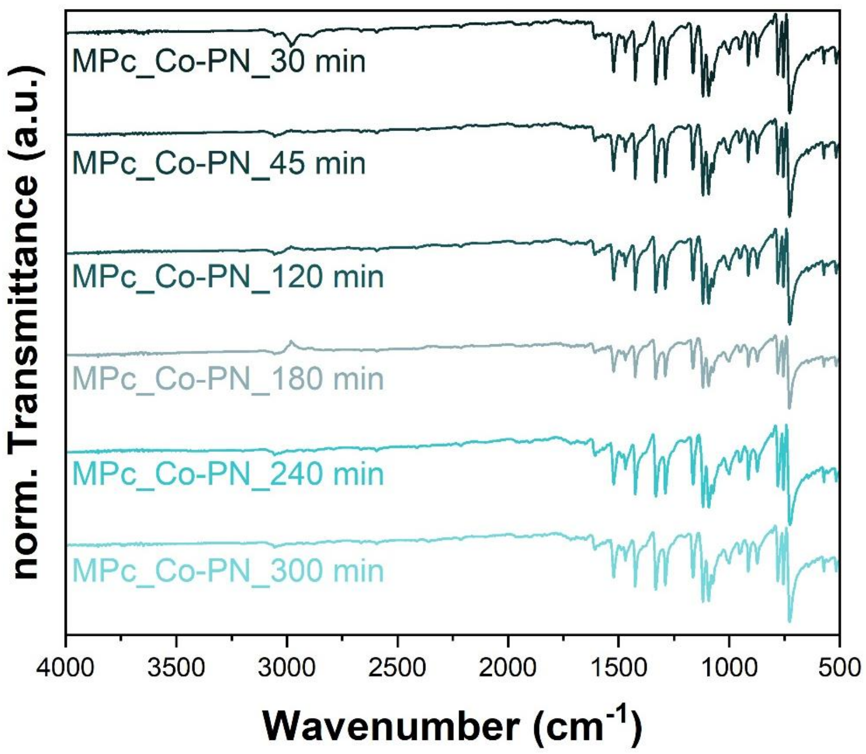


**Figure S9**: FTIR spectroscopy of the obtained cobalt phthalocyanines of the time screening.

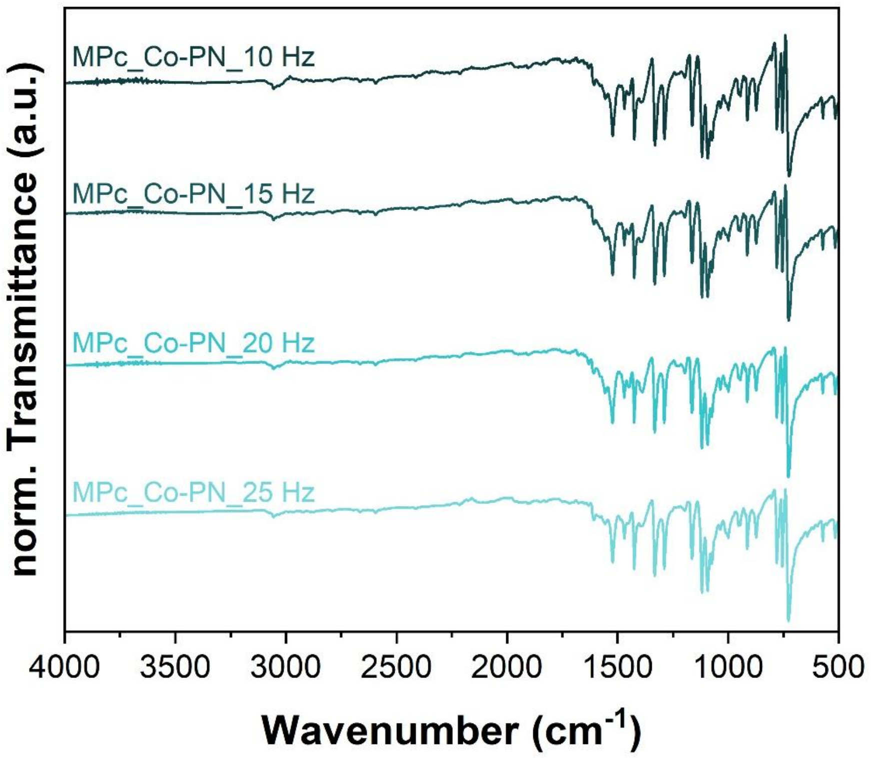


**Figure S10**: FTIR spectroscopy of the obtained cobalt phthalocyanines of the frequency screening.

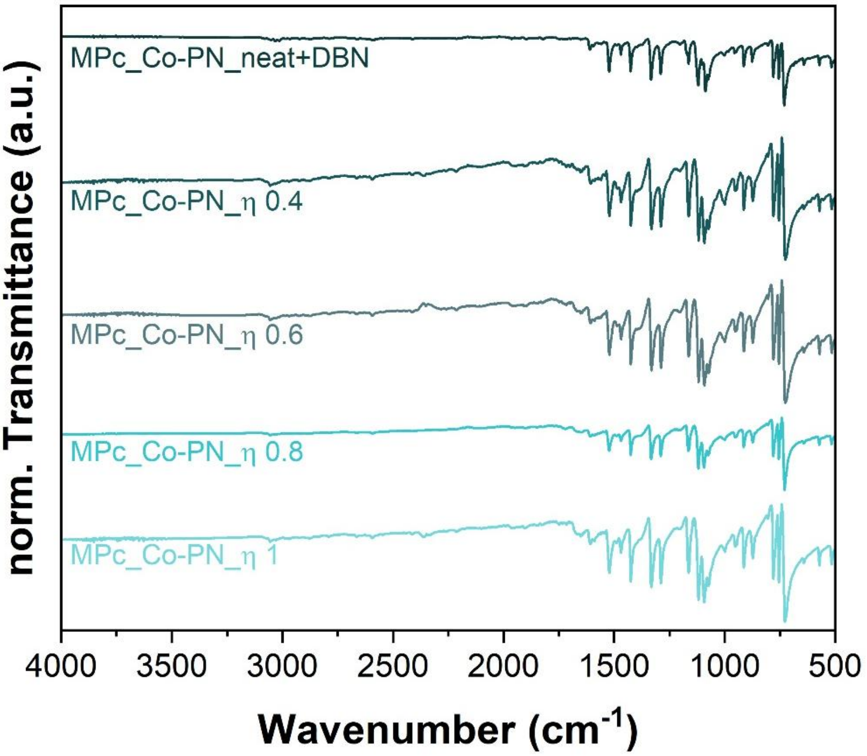


**Figure S11**: FTIR spectroscopy of the obtained cobalt phthalocyanines of the liquid-assisted grinding screening.

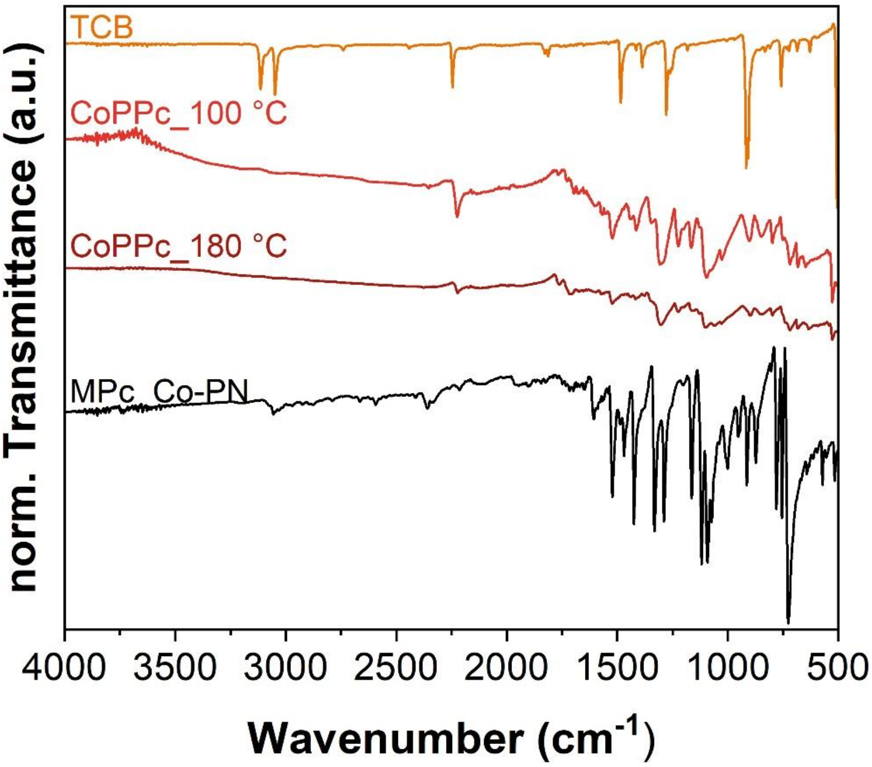


**Figure S12**: FTIR spectroscopy of the obtained cobalt polyphthalocyanine.

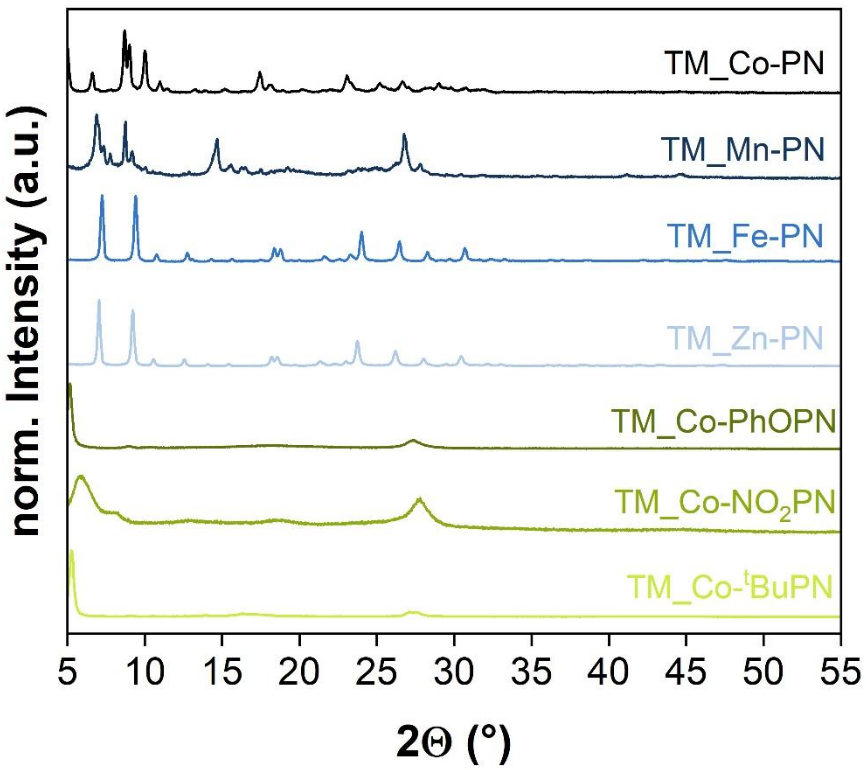


**Figure S13**: The powder x-ray diffractions of the obtained metal phthalocyanines.

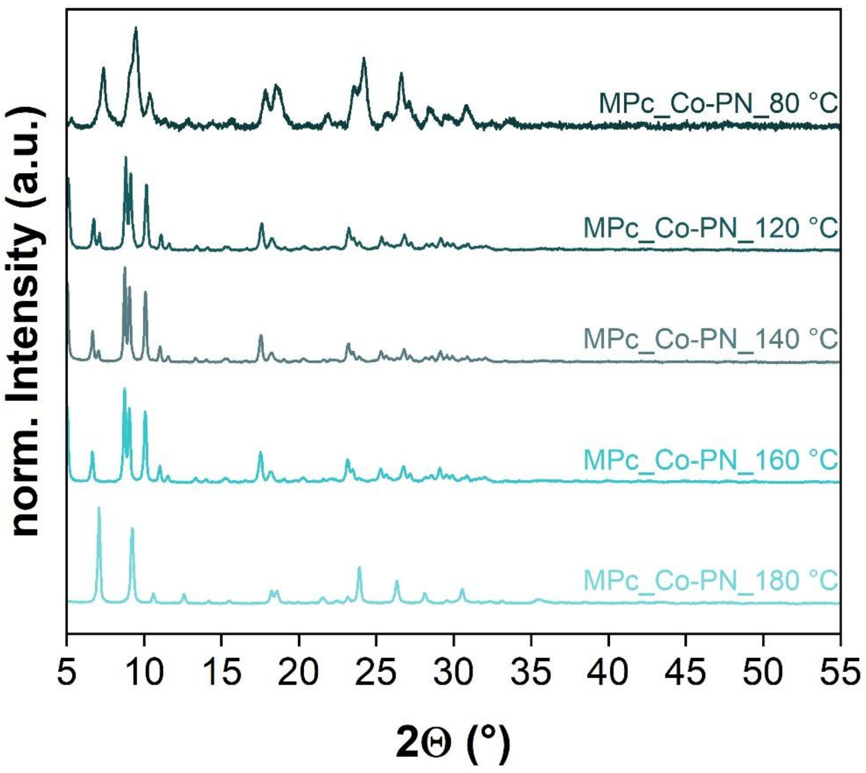


**Figure S14**: The powder x-ray diffractions of the obtained cobalt phthalocyanines of the temperature screening.

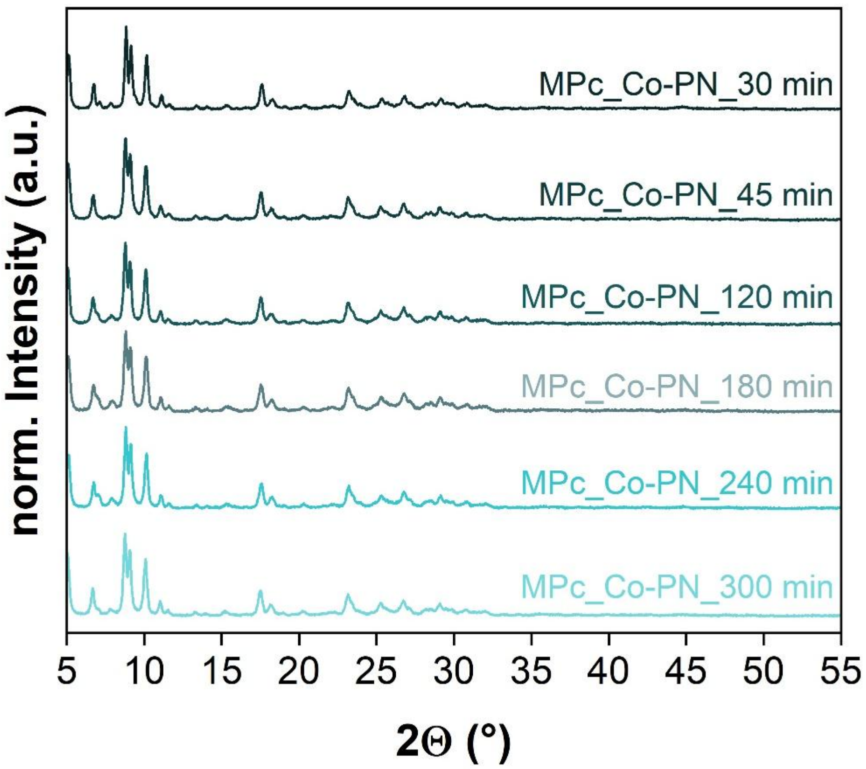


**Figure S15**: The powder x-ray diffractions of the obtained cobalt phthalocyanines of the time screening.

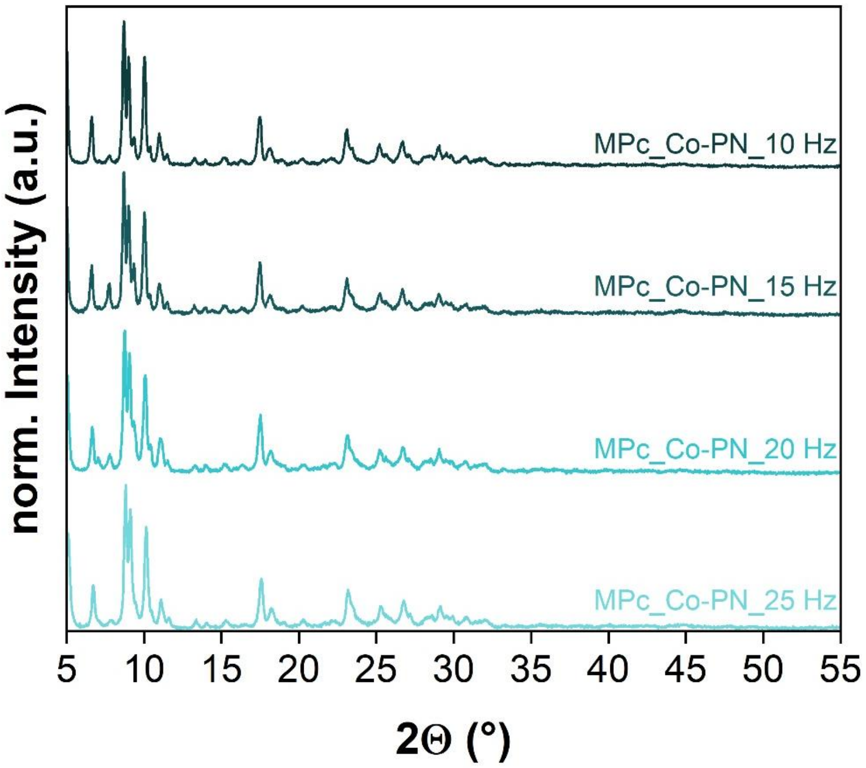


**Figure S16**: The powder x-ray diffractions of the obtained cobalt phthalocyanines of the frequency screening.

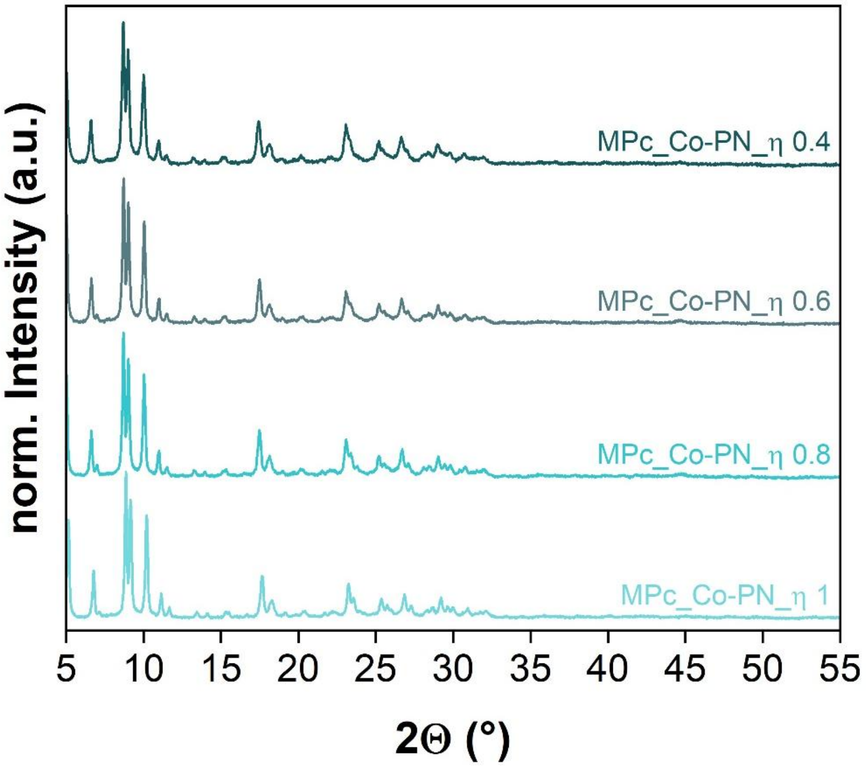


**Figure S17**: The powder x-ray diffractions of the obtained cobalt phthalocyanines of the liquid-assisted grinding screening.

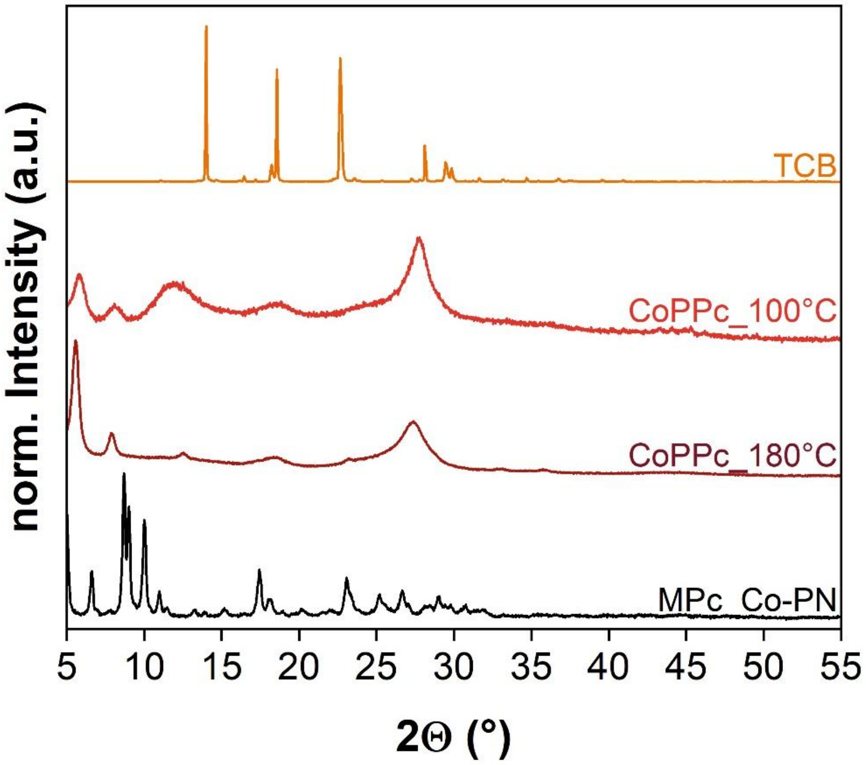


**Figure S18**: The powder x-ray diffractions of the obtained cobalt polyphthalocyanine.

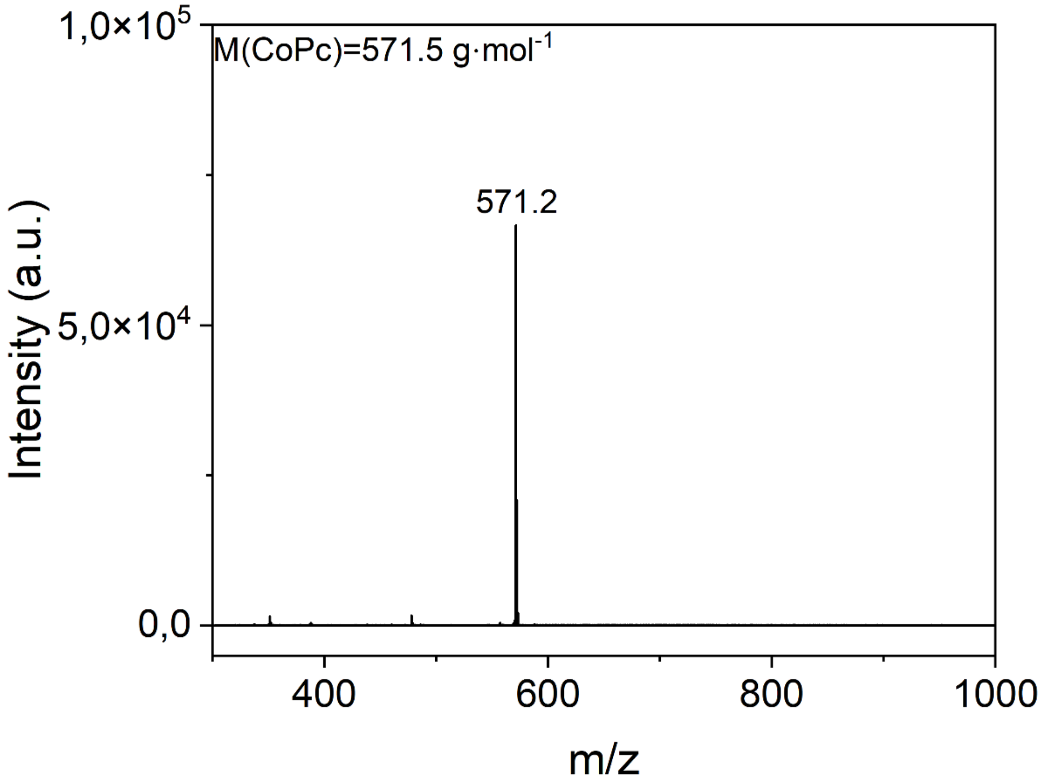


**Figure S19**: MALDI-TOF mass spectrum of the obtained cobalt phthalocyanine.

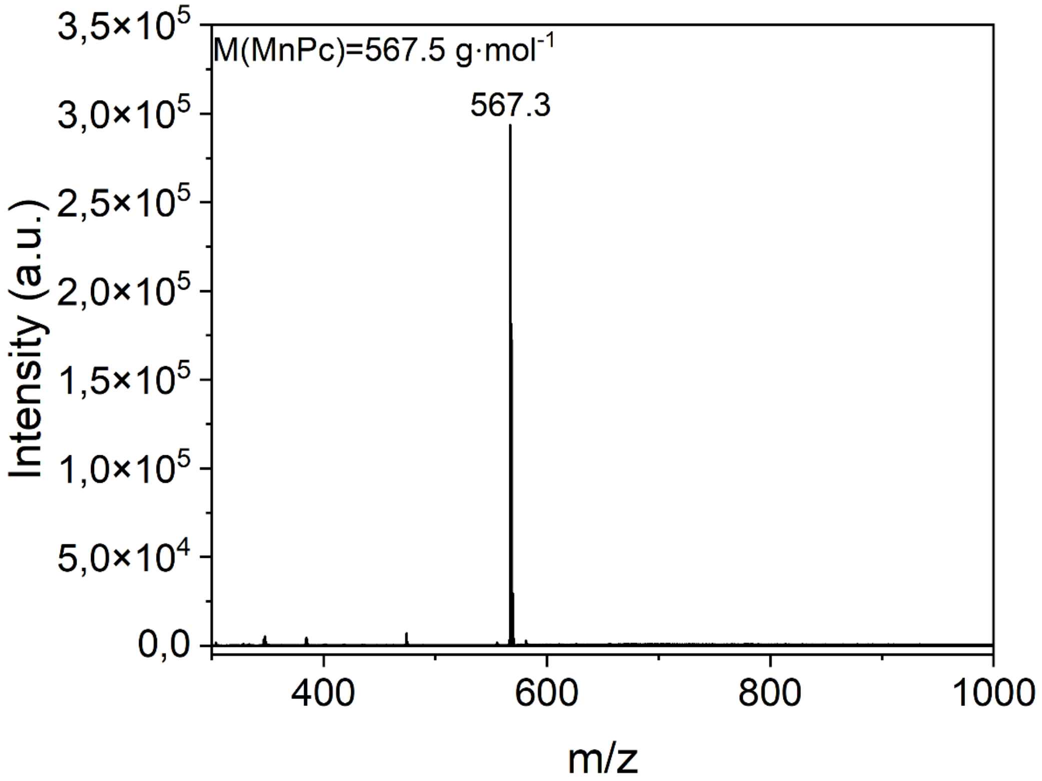


**Figure S20**: MALDI-TOF mass spectrum of the obtained manganese phthalocyanine.

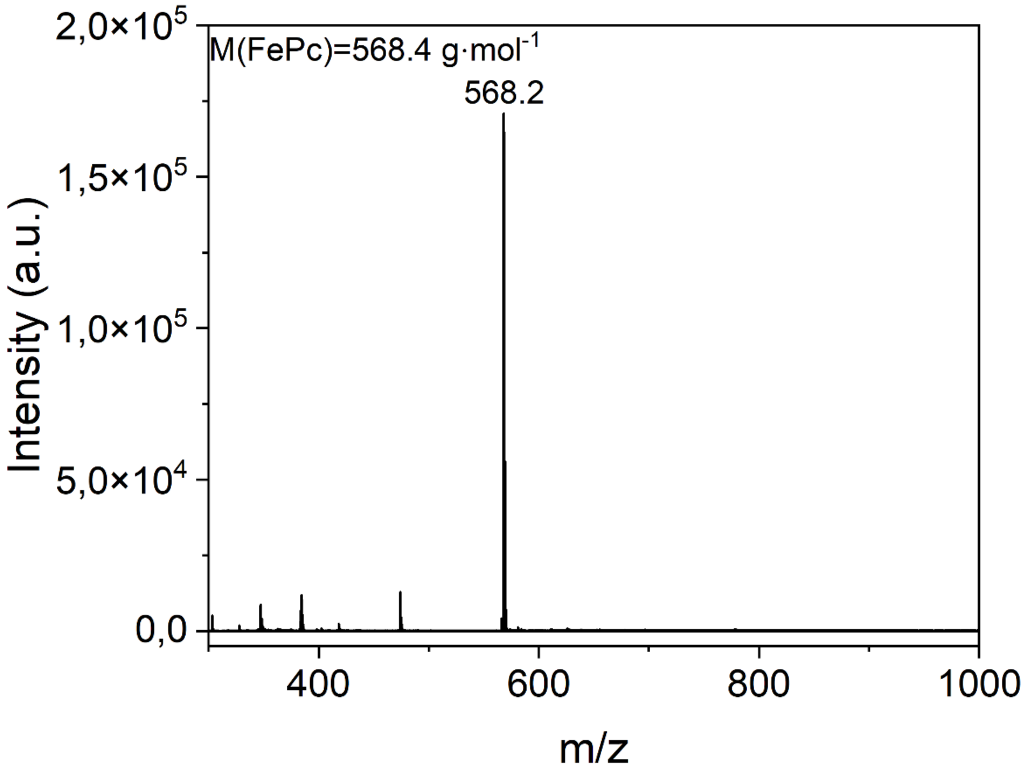

**Figure S21**: MALDI-TOF mass spectrum of the obtained iron phthalocyanine.

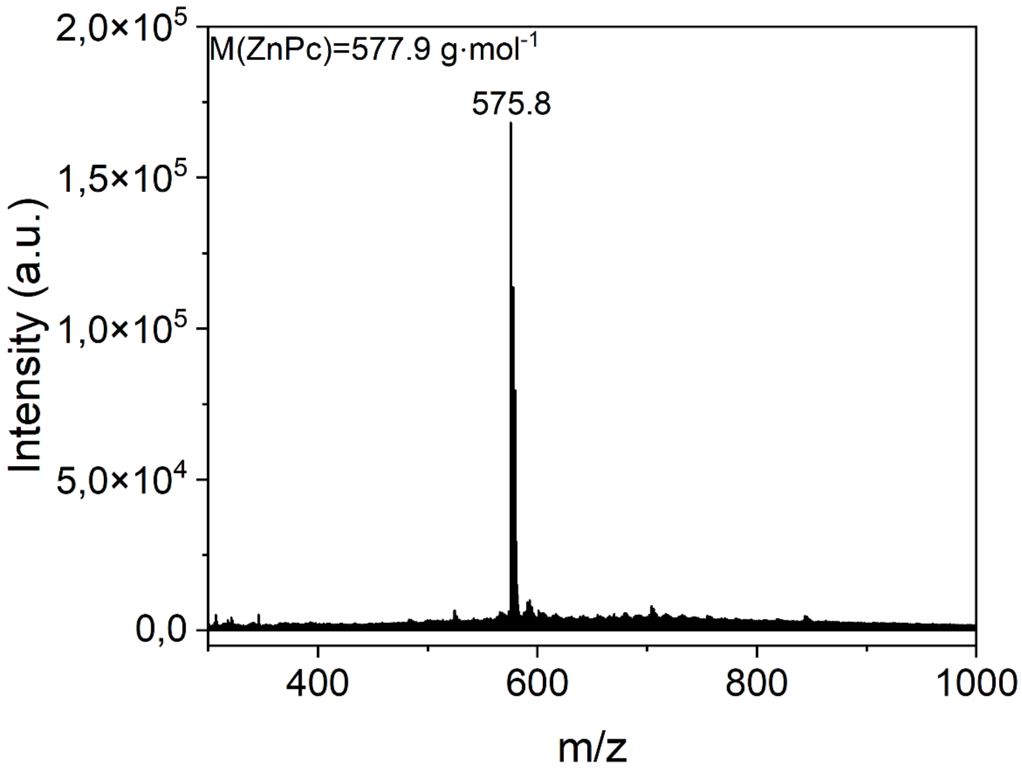

**Figure S22**: MALDI-TOF mass spectrum of the obtained zinc phthalocyanine.

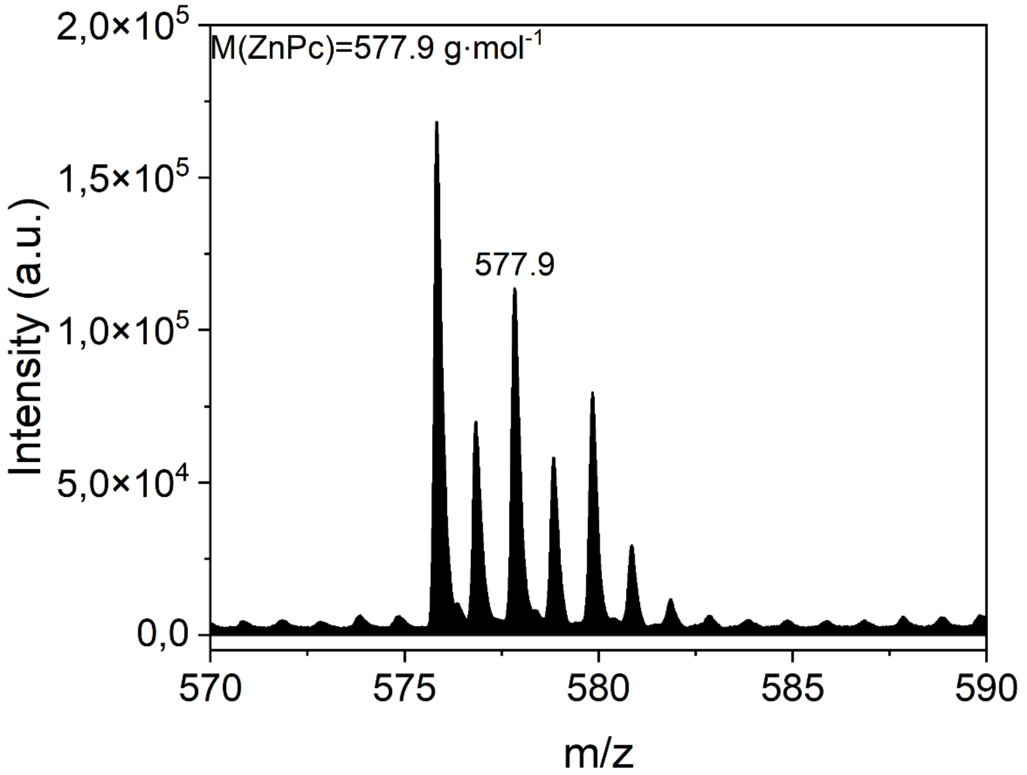


**Figure S23**: Zoomed MALDI-TOF mass spectrum with the pattern of the obtained zinc phthalocyanine.

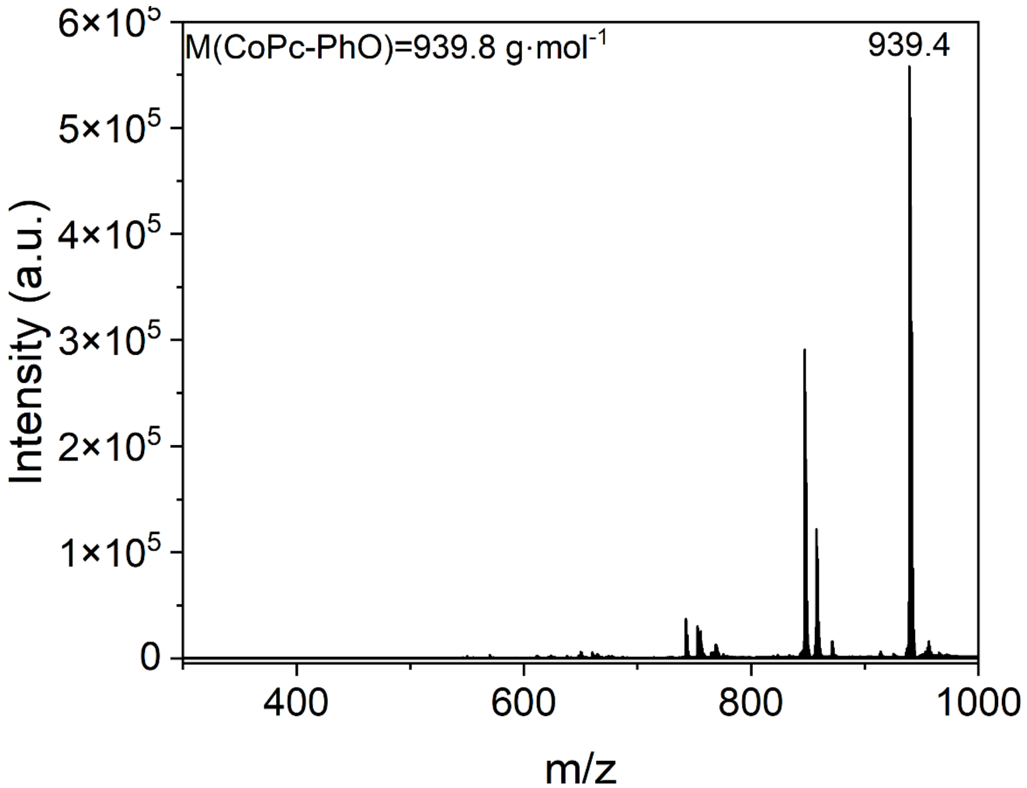


**Figure S24**: MALDI-TOF mass spectrum of the obtained cobalt phthalocyanines using 4-Phenoxyphthalonitrile.

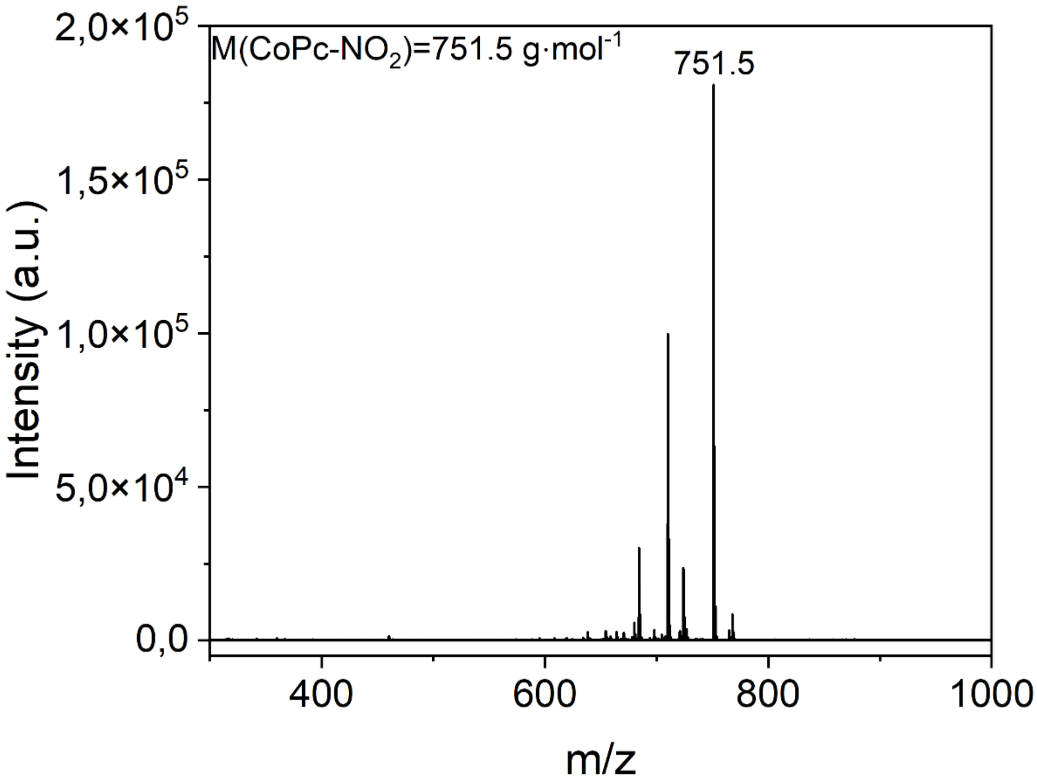


**Figure S25**: MALDI-TOF mass spectrum of the obtained cobalt phthalocyanine using 4-Nitrophthalonitrile.

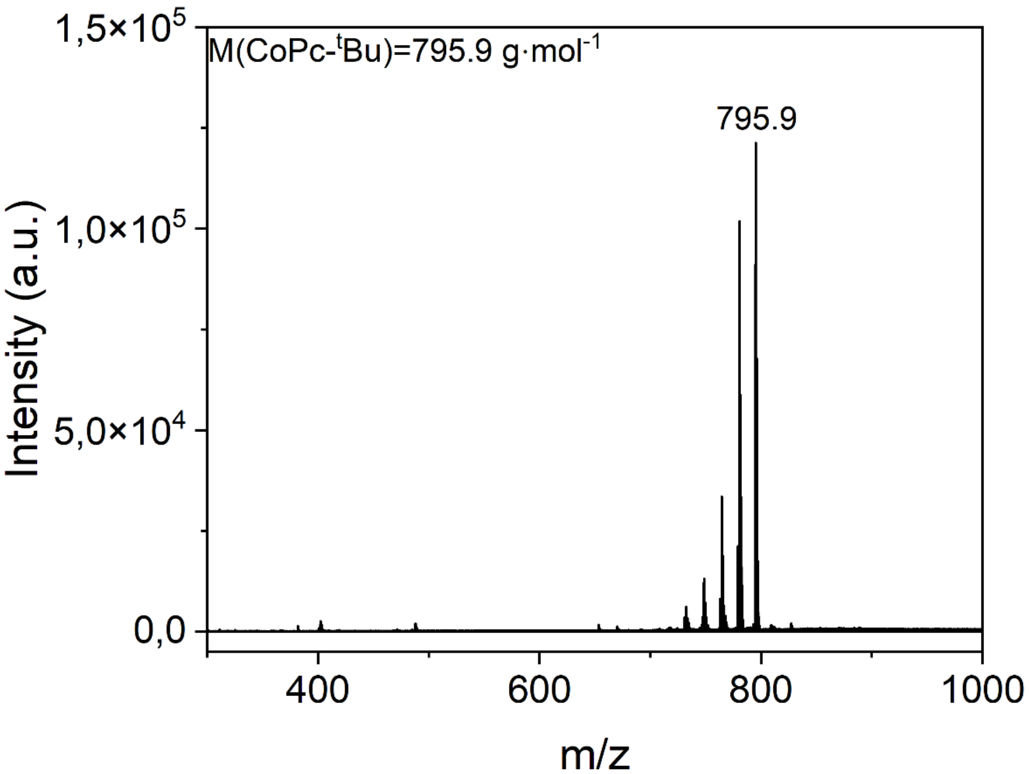


**Figure S26**: MALDI-TOF mass spectrum of the obtained cobalt phthalocyanine using 4-(tert-Butyl)phthalonitrile.

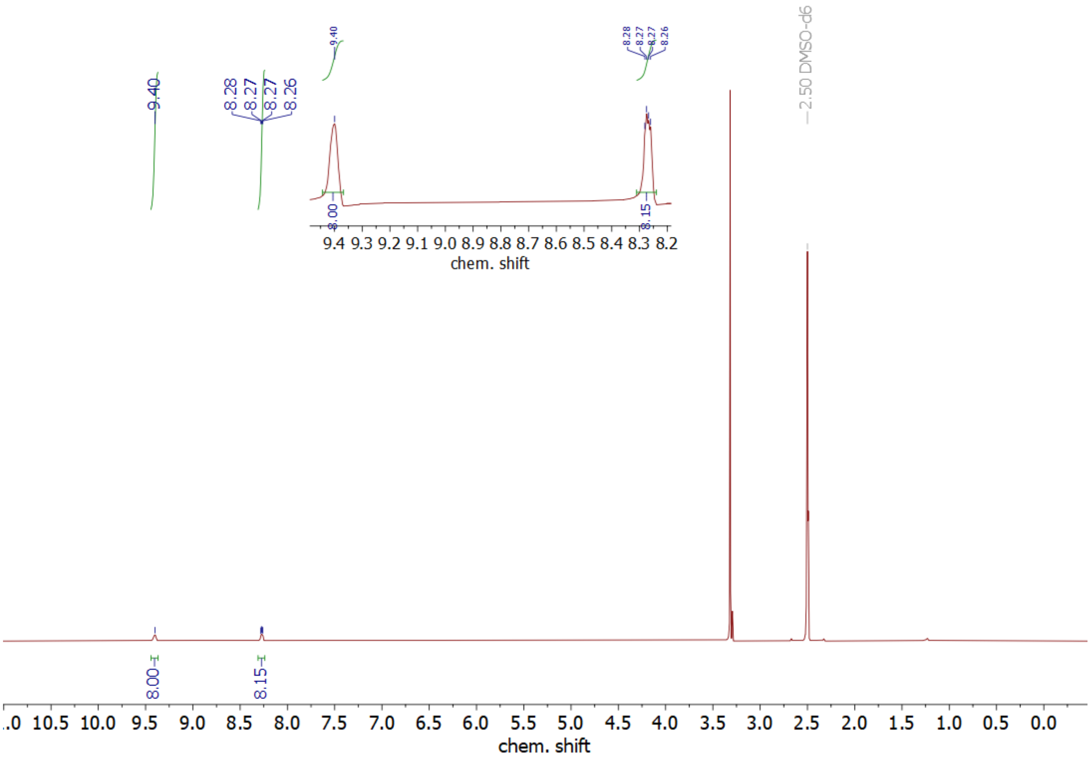


**Figure S27**: $^{1}$H NMR spectrum of the obtained zinc phthalocyanine illustrating to peaks with an integral of each 8 protons (9.44-9.37 ppm, 8.31-8.28 ppm).

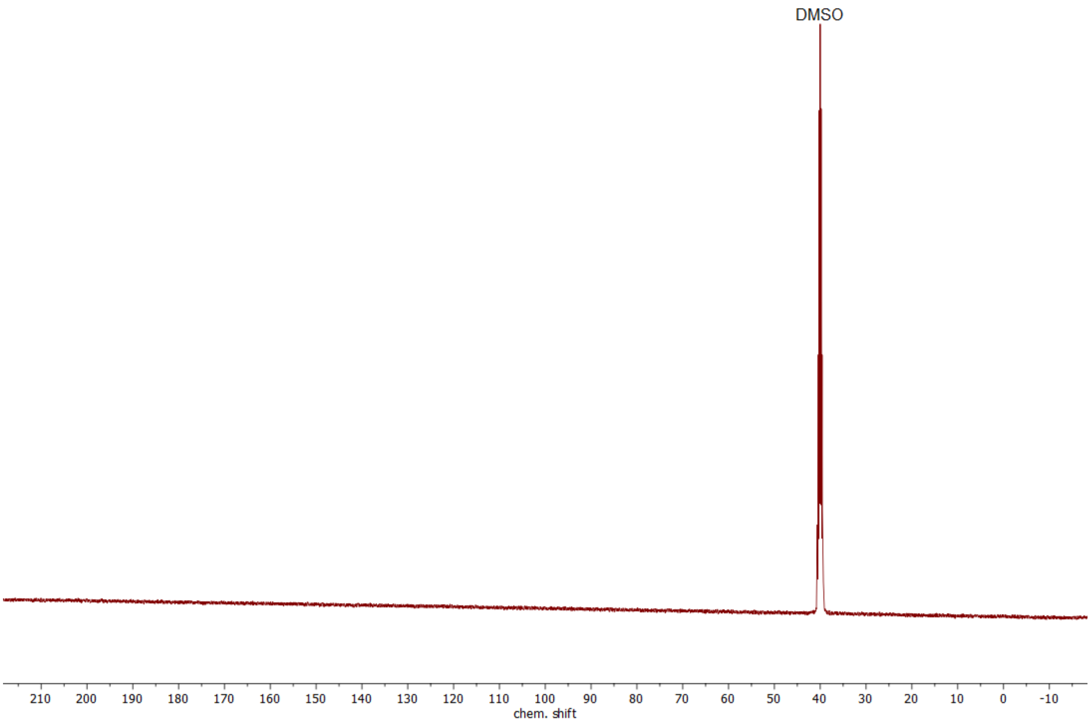


**Figure S28**: $^{13}$C NMR spectrum of the obtained zinc phthalocyanine illustrating no peaks due to the low solubility of the product.

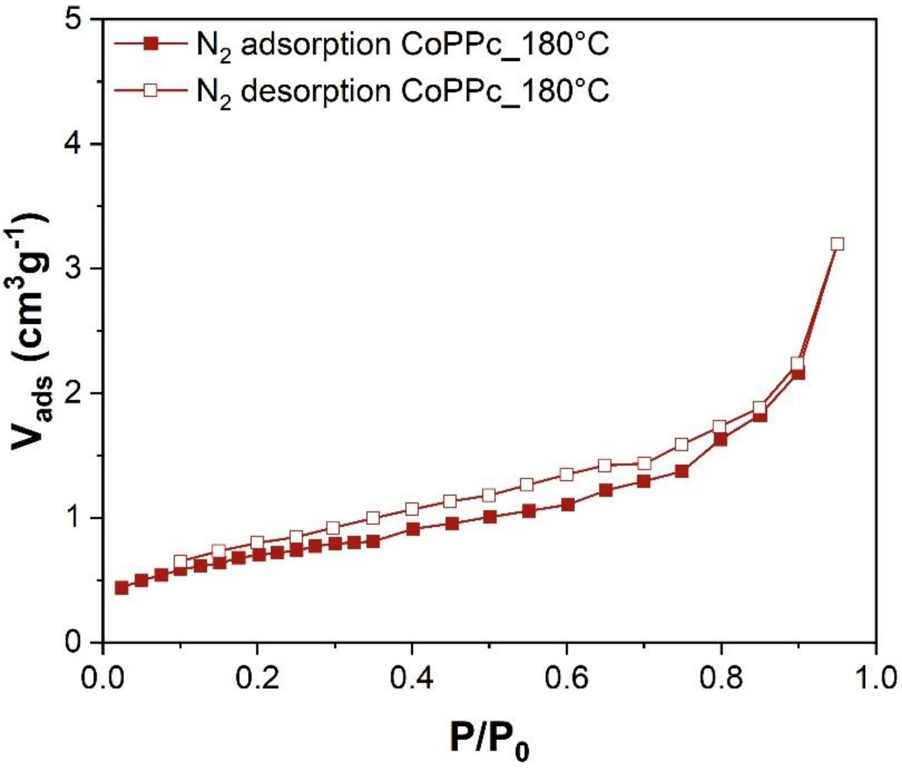


**Figure S29**: Nitrogen physisorption measurement of CoPPc_180°C displays a type 2 isotherm with a specific surface area of 3 $m^2g^{-1}$.

# 5. Supplementary Figures

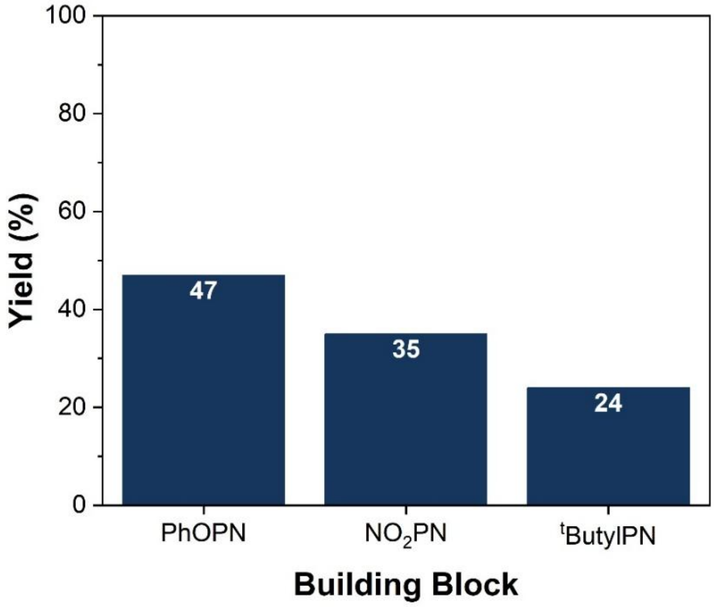


**Figure S30**: Screening of the building blocks 4-phenoxyphthalonitrile (MPC_Co-PhOPN_m), 4-nitrophthalonitrile (MPC_Co-$NO_2$PN_m) and 4-(tert-butyl)phthalonitrile (MPC_Co-$^t$ButylPN_m) at the milder conditions 100 °C for 60 min.

# 6. Green Chemistry Metrics

**Table S2**: Green metrics and their formulas which were used.[1]

| Green metric | Abbreviation | Formula | Optimal value |
|---|---|---|---|
| **Environmental factor** | E-factor | $E - factor = \frac{\sum m(Waste)}{m(Product)}$ | 0 |
| **EcoScale** | - | $EcoScale = 100 - Penalty\ points$ | 100 |
| **Mass Intensity** | MI | $MI = \frac{Total\ mass\ input\ materials\ excluding\ water}{m(Product)}$ | 1 |

## 6.1. E-Factor

*Calculation thermo-mechanochemical approach (incl. workup):*

**Table S3**: Used chemicals and their masses for the E-Factor calculation of the thermo-mechanochemical approach.

| Chemical | Mass (g) |
|---|---|
| DBN | 0.0695 |
| DMF | 0.197 |
| $H_2O$ | 49.9 |
| Acetone | 39.5 |
| CoPc | 1.0968 |

$$E - Factor = \frac{(0.0695 + 0.197 + 49.9 + 39.5)g}{1.0968g} = 81.8$$

*Calculation thermo-mechanochemical approach (excl. water):*

**Table S4**: Used chemicals and their masses for the E-Factor calculation of the thermo-mechanochemical approach, excluding water.

| Chemical | Mass (g) |
|---|---|
| DBN | 0.0695 |
| DMF | 0.197 |
| Acetone | 39.5 |
| CoPc | 1.0968 |

$$E - Factor = \frac{(0.0695 + 0.197 + 39.5)g}{1.0968g} = 36.3$$

*Calculation thermos-mechanochemical approach (excl. workup):*

**Table S5**: Used chemicals and their masses for the E-Factor calculation of the thermo-mechanochemical approach excluding workup.

| Chemical | Mass (g) |
|---|---|
| DBN | 0.0695 |
| DMF | 0.197 |
| CoPc | 1.0968 |

$$E - Factor = \frac{(0.0695 + 0.197)g}{1.0968g} = 0.2$$

## 6.2. Mass intensity

*Calculation thermo-mechanochemical approach:*

**Table S6**: Used chemicals and their masses for the mass intensity calculation of the thermo-mechanochemical approach.

| Chemical | Mass (g) |
|---|---|
| Phthalonitrile | 1.03 |
| Co(II)acetat | 0.354 |
| DBN | 0.0695 |
| DMF | 0.197 |
| Acetone | 39.5 |
| CoPc | 1.0968 |

$$MI = \frac{(1.03 + 0.354 + 0.0695 + 0.197 + 39.5)g}{1.0968g} = 37.5$$

*Calculation DES approach[2,3]:*

**Table S7**: Used chemicals and their masses for the mass intensity calculation of the DES approach.

| Chemical | Mass (g) |
|---|---|
| Phthalonitrile | 0.256 |
| Co(II)chlorid | 0.065 |
| Cholin chloride/Urea (DES) | 2.5 |
| Acetone | 7.9 |
| Sulfuric acid | 9.15 |
| CoPc | 0.206 |

$$MI = \frac{(0.256 + 0.065 + 2.5 + 7.9 + 9.15)g}{0.206g} = 96.5$$

*Calculation benign solvent approach[4]:*

**Table S8**: Used chemicals and their masses for the mass intensity calculation of the benign solvent approach.

| Chemical | Mass (g) |
|---|---|
| Phthalonitrile | 1.0 |
| $Co(OAc)_2 \cdot 4H_2O$ | 0.583 |
| DBU | 0.032 |
| glycerol/anisol mix | 5.5 |
| HCl | 15.15 |
| Methanol | 23.76 |
| CoPc | 0.802 |

$$MI = \frac{(1.0 + 0.583 + 0.032 + 5.5 + 15.15 + 23.76)g}{0.802g} = 57.4$$

## 6.3. EcoScale

**Table S9**: Calculation of the EcoScale of the thermos-mechanochemical approach, the deep eutectic solvent approach[3] and the benign solvent method[4].

| Parameter | Penalty | Thermo-mechanochemical | | Deep eutectic solvent (DES) | | Benign solvent | |
|---|---|---|---|---|---|---|---|
| 1. **Yield** | (100-%yield)/2 | 93 | 3.5 | 72 | 14 | 72 | 14 |
| 2. **Price of reaction components** (to obtain 10 mmol of product)<br>• Inexpensive (<\$10)<br>• Expensive (>\$10 and <\$50)<br>• Very expensive (>\$50) | 0<br>3<br>5 | | 0 | | 0 | | 0 |
| 3. **Safety**<br>• N (dangerous for environment)<br>• T (toxic)<br>• F (highly flammable)<br>• E (explosive)<br>• F+ (extremely flammable)<br>• T+ (extremely toxic) | 5<br>5<br>5<br>10<br>10<br>10 | Phthalonitrile (T)<br>$Co(OAc)_2$ (T)<br>DMF (T)<br>Acetone (F) | 5<br>5<br>5<br>5 | Phthalonitrile (T)<br>$CoCl_2$ (T)<br>Acetone (F) | 5<br>5<br>5 | Phthalonitrile (T)<br>$Co(OAc)_2 \cdot 4H_2O$ (T)<br>Methanol (T, F) | 5<br>5<br>10 |
| 4. **Technical setup**<br>• Common setup<br>• Funnel, syringe pump, gas pressure regulator<br>• Unconventional activation technique<br>• Pressure equipment, >1 atm<br>• Any additional glassware<br>• (Inert)gas atmosphere<br>• Glovebox | 0<br>1<br>2<br>3<br>1<br>1<br>3 | Ball mill with heating jackets<br>Glovebox | 2<br>3 | Common setup | 0 | Common setup | 0 |
| 5. **Temperature/time**<br>• Room temperature, <1 h<br>• Room temperature, <1 h<br>• Heating, <1 h<br>• Heating, >1 h<br>• Cooling to 0 °C<br>• Cooling, <0 °C | 0<br>1<br>2<br>3<br>4<br>5 | Heating, >1 h | 3 | Heating, <1 h | 2 | Heating, >1 h | 3 |
| 6. **Workup and purification**<br>• None<br>• Cooling to room temperature<br>• Adding solvents<br>• Simple filtration<br>• Removal of solvent with bp <150 °C<br>• Crystallization and filtration<br>• Removal of solvent with bp >150 °C<br>• Solid phase extraction<br>• Distillation<br>• Sublimation<br>• Liquid-liquid extraction or washing<br>• Classical chromatography | 0<br>0<br>0<br>0<br>0<br>1<br>2<br>2<br>3<br>3<br>3<br>10 | Adding water<br>Adding acetone<br>Washing | 0<br>0<br>3 | Adding water<br>Filtration<br>Washing | 0<br>0<br>3 | Cooling to r.t.<br>Adding HCl 1N<br>Washing | 0<br>0<br>3 |
| 7. **Total** | 100 | 34.5 | | 34 | | 40 | |
| 8. **EcoScale** | 100-penalties | 65.5 | | 66 | | 60 | |

## 6.4. Comparison Green Merics

**Table S10**: Comparison of the green metrics of the different methods.

| Green metrics | Optimal value | Thermo-mechanochemical | Deep eutectic solvent | Benign solvent |
|---|---|---|---|---|
| **E-Factor** | 0 | 81.8 | 958.4[5] | 105.3[5] |
| **E-Factor without water** | 0 | 36.3 | 83.5[5] | 55.5[5] |
| **Mass intensity** | 1 | 37.5 | 96.5 | 57.4 |
| **EcoScale** | 100 | 65.5 | 66 | 60 |

# 7. Author contributions

| | |
|---|---|
| Stefanie Hutsch | Preparation of the manuscript, mechanochemical reference and polymer reactions and sample purification, Raman, PXRD and Physisorption measurements, Green Metrics analysis. |
| Malte Niewind | Mechanochemical reactions and sample purification, NMR, IR and Raman measurements, reviewed the manuscript. |
| Sven Grätz | Supervision of the project. |
| Lars Borchardt | Finalization of the manuscript and supervision of project. |

# 8. References


[1] a) A. D. Curzons, D. N. Mortimer, D. J. C. Constable, V. L. Cunningham, *Green Chem.* **2001**, *3*, 1; b) N. Fantozzi, J.-N. Volle, A. Porcheddu, D. Virieux, F. García, E. Colacino, *Chemical Society reviews* **2023**, *52*, 6680; c) D. J. C. Constable, A. D. Curzons, V. L. Cunningham, *Green Chem* **2002**, *4*, 521; d) K. van Aken, L. Strekowski, L. Patiny, *Beilstein journal of organic chemistry* **2006**, *2*, 3.

[2] Q. Zhang, K. de Oliveira Vigier, S. Royer, F. Jérôme, *Chemical Society reviews* **2012**, *41*, 7108.

[3] A. Shaabani, S. E. Hooshmand, R. Afshari, S. Shaabani, V. Ghasemi, M. Atharnezhad, M. Akbari, *Journal of Solid State Chemistry* **2018**, *258*, 536.

[4] G. Zanotti, P. Imperatori, A. M. Paoletti, G. Pennesi, *Molecules (Basel, Switzerland)* **2021**, *26*.

[5] G. Zanotti, F. Palmeri, V. Raglione, *Chemistry (Weinheim an der Bergstrasse, Germany)* **2024**, *30*, e202400908.
